# The relationship between academic self-efficacy and test anxiety: a meta-analysis of Chinese secondary school students

**DOI:** 10.3389/fpsyg.2026.1891963

**Published:** 2026-09-23

**Authors:** Weiyi He

**Affiliations:** Graduate School Student, College of Sciences, Shihezi University, Shihezi, Xinjiang, China

**Keywords:** academic self-efficacy, control-value theory, meta-analysis, secondary school students, social cognitive theory, test anxiety

## Abstract

Existing empirical studies report inconsistent correlation coefficients, ranging from −0.105 to −0.792, for the association between academic self-efficacy and test anxiety among Chinese secondary school students. Although evidence indicates that academic self-efficacy predicts test anxiety more strongly than domain-general self-efficacy (global confidence across all life domains), most prior meta-analyses have focused on the latter rather than on academic self-efficacy (perceived competence in specific academic domains). Furthermore, few quantitative meta-analyses have systematically decomposed moderators contributing to cross-study heterogeneity, leaving a notable research gap. Literature was retrieved from CNKI and Web of Science. The present meta-analysis included 30 studies (*N* = 27,865) of Chinese secondary school students. Publication bias was evaluated via multiple complementary methods, including Egger’s linear regression test, Duval and Tweedie’s trim-and-fill, classic fail-safe N, and Begg and Mazumdar’s rank correlation test. Grounded in social cognitive theory and control-value theory, this study aimed to estimate the overall correlation magnitude and test four potential moderators via subgroup analysis and Knapp-Hartung-corrected mixed-effects meta-regression: grade level, geographic region, academic self-efficacy measurement instruments, and test anxiety measurement instruments. Random effects models based on Fisher’s Z transformation revealed a significant moderate negative correlation (*r* = −0.359, 95% CI [−0.405, −0.312]). Subgroup analyses and meta-regression indicated non-significant moderating effects for grade level, geographic region, and test anxiety measurement instruments, whereas academic self-efficacy measurement instruments significantly moderated the association. However, this moderating effect should be treated as preliminary suggestive evidence. Some subgroups comprised only one or two studies, so results should be interpreted with caution. Theoretically, this study is the first large-sample meta-analysis on this topic among Chinese secondary school students, providing synthesized empirical evidence for research on adolescent academic emotions and enriching the validation of social cognitive theory and control-value theory in the Chinese context. Practically, it offers tentative, hypothesis-generating guidance for selecting academic self-efficacy measurement instruments and proposes potential intervention directions for teachers, school counselors, and educational policymakers.

## 1 Introduction

Mental health and subjective well-being among adolescents have become key concerns in educational psychology. Anxiety disorders are particularly prevalent among adolescents, with rising prevalence rates documented in recent decades (Du et al., 2025; Bie et al., 2024). Test anxiety is a specific form of anxiety experienced in academic assessment situations.

Test anxiety is a widespread academic psychological problem around the world; Lohiya et al. used the Test Anxiety Inventory (TAI) and k-means cluster analysis to identify low, moderate, and high anxiety subgroups, and they reported that 18% of Indian students aged 8–18 had severe test anxiety while 48% had moderate test anxiety (Lohiya et al., 2021); with the Revised Test Anxiety scale (RTA) and item-response anchors, Putwain and Daly found that 16.4% of UK secondary school students were in the high test anxiety category (Putwain and Daly, 2014); Huang and Zhou used the Chinese revised Test Anxiety scale (TAS) and a cut-off score above 20, and their results showed that about 29.8% of Chinese secondary school students met the standard for severe test anxiety (Huang and Zhou, 2019); similar prevalence rates have also been documented in other countries (Valls et al., 2025).

Test anxiety is a multifaceted construct encompassing cognitive, physiological, and affective reactions elicited when individuals anticipate exam failure or perceive negative evaluation as threatening (Putwain et al., 2021). Empirical evidence indicates that excessive test anxiety undermines adolescents’ physical and mental health as well as academic functioning. Emotionally, it reduces school well-being and increases the risk of clinical emotional disorders (Putwain et al., 2021). Academically, it undermines learning motivation (Putwain et al., 2015), impairs cognitive processing efficiency (Duan et al., 2015), and ultimately reduces academic performance (Von der Embse et al., 2018; Putwain et al., 2015).

Self-efficacy is a critical protective factor against test anxiety, and consistent evidence shows a significant negative correlation between them (Brandmo et al., 2018); Pajares separated general self-efficacy from academic self-efficacy, and showed that domain-specific academic self-efficacy predicts negative academic emotions like test anxiety more strongly than general self-efficacy (Pajares, 1996); more evidence also shows that academic self-efficacy is significantly and negatively linked to the strength of students’ test anxiety (Lei et al., 2021).

Cross-cultural comparisons reveal psychological differences between adolescents in Confucian heritage cultures and Western cultures. East Asian secondary school students tend to report greater test anxiety and lower academic self-confidence than their Western counterparts (Morony et al., 2013). China operates a high-stakes, exam-oriented selection system focused on entrance examinations at both the senior high school and college levels (Wu et al., 2025), whereas many Western education systems feature greater external differentiation and vocational orientation, with multiple academic and vocational tracks (Bol and Van de Werfhorst, 2013).

A systematic review of existing Chinese empirical studies reveals two main limitations; first, individual primary studies usually report bivariate correlations without combining them quantitatively, the reported correlation values differ greatly, and the causes of differences across studies are still not fully explored; second, measurement instruments are not standardized, because adapted Western scales are mixed with questionnaires developed in China, which may bring systematic bias; furthermore, no agreement exists on whether demographic factors like grade and region and methodological features like measurement instruments influence this relationship.

Against this background, the present study uses a meta-analytical method to combine empirical studies on Chinese secondary school students; it calculates the overall correlation between academic self-efficacy and test anxiety, and examines whether four factors—grade level, geographic region, academic self-efficacy measurement instruments, and test anxiety measurement instruments—affect this relationship.

## 2 Theoretical foundations

### 2.1 Social cognitive theory

Bandura’s social cognitive theory provides the core framework for unpacking the mechanism linking academic self-efficacy to test anxiety (Bandura, 1978). The theory’s triadic reciprocal determinism posits bidirectional interactions among individual cognition, overt behavior, and external environmental stimuli. Distinct from general self-efficacy, academic self-efficacy represents a domain-specific efficacy belief specific to academic contexts, defined as students’ subjective judgments of their capacity to complete scholastic tasks and attain academic goals.

Academic self-efficacy regulates individuals’ affective appraisal and stress reactivity. Students with high academic self-efficacy interpret examination scenarios positively and are unlikely to construe tests as threatening, thereby experiencing less tension, worry, and test anxiety. Conversely, students with low academic self-efficacy tend to anticipate exam failure and perceive testing as an intense stressor, which precipitates chronic test anxiety (Bandura, 1978; Robson et al., 2023). Bandura (1978) also identifies physiological and affective states as one of four primary sources of self-efficacy. Palpitations, nervousness, and other somatic symptoms induced by test anxiety further erode students’ efficacy judgments, creating a reciprocal cycle: diminished academic self-efficacy leads to elevated test anxiety, which further undermines academic self-efficacy. This cyclical dynamic explains the psychological logic underpinning the stable negative correlation between the two variables.

### 2.2 Control-value theory

Pekrun’s control-value theory provides a framework for explaining how academic self-efficacy operates within learning and evaluative contexts to shape academic emotions (Pekrun, 2006). The theory posits that the emergence of pleasant or maladaptive academic emotions hinges on two interrelated cognitive appraisals: control appraisal and value appraisal. When individuals perceive themselves as capable of regulating academic tasks and anticipated learning outcomes, positive academic emotions emerge. In contrast, maladaptive academic emotions predominate when learners view academic objectives as unattainable and experience a diminished sense of control.

As a prototypical maladaptive academic emotion (Pekrun, 2006), test anxiety is readily explained by control-value theory. Academic self-efficacy forms the basis of students’ control appraisals of scholastic performance. Low academic self-efficacy corresponds to a weak sense of agency over exam outcomes and heightened anticipation of failure; when combined with students’ high subjective valuation of examinations, intense test anxiety arises. Consequently, control-value theory accounts for the negative association between academic self-efficacy and test anxiety.

## 3 *A priori* theoretical expectations and research questions

### 3.1 Overall association between academic self-efficacy and test anxiety

Students who perceive greater control over academic challenges may appraise testing situations as less threatening, which could be associated with diminished anxiety responses. Conversely, students who doubt their academic capabilities may experience heightened threat perception in evaluative contexts. Based on this reasoning, a negative association between academic self-efficacy and test anxiety is expected, though its magnitude remains an empirical question to be quantified by the present meta-analysis.

### 3.2 Grade level moderation of the association

Proximity to high-stakes entrance examinations raises subjective value appraisals of exam outcomes, suggesting that the negative correlation may be stronger among senior high school students facing the college entrance examination. Grade level moderation is therefore treated as an exploratory empirical question.

### 3.3 Geographic region moderation of the association

Chinese geographic subgroups differ in educational resources and the intensity of academic competition. Theoretically, such contextual disparities may alter students’ perceived control and subjective value appraisals regarding examinations. In Eastern regions with abundant educational resources and fierce academic competition, students attach high subjective value to examination outcomes. Students with low academic self-efficacy are more prone to loss of control amid fierce competition and consequently experience higher test anxiety, leading to a stronger negative correlation between the two variables. Geographic region moderation is therefore treated as an exploratory empirical question.

### 3.4 Moderation by academic self-efficacy measurement instruments

Academic self-efficacy exhibits domain-specific properties: efficacy beliefs tied to concrete academic contexts possess stronger predictive power for corresponding academic emotion outcomes than broad, decontextualized efficacy beliefs. Academic self-efficacy measurement instruments that heavily weight examination-relevant task content directly tap efficacy perceptions tied to test-taking scenarios and should therefore produce stronger negative correlations with test anxiety. By comparison, academic self-efficacy measurement instruments focused predominantly on general daily learning content, with limited examination-focused item coverage, would yield weaker associations. Consequently, the type of academic self-efficacy measurement instrument is expected to moderate the magnitude of the pooled correlation. This expectation is exploratory.

### 3.5 Moderation by test anxiety measurement instruments

According to control-value theory, test anxiety is a multidimensional construct comprising cognitive worry, physiological-affective arousal, and behavioural avoidance responses, which arise from the joint operation of control and value appraisals (Pekrun, 2006). Existing self-report test anxiety instruments differ considerably in the weight assigned to these three dimensions within their item pools. Academic self-efficacy primarily reflects individuals’ cognitive judgments of their own scholastic competence; as such, it should exert the strongest predictive power for the cognitive worry facet of test anxiety. In contrast, its predictive capacity is comparatively weaker for physiological anxiety symptoms that are less closely linked to perceived competence. Accordingly, test anxiety measurement instruments are expected to moderate the magnitude of the correlation with academic self-efficacy: measurement instruments emphasising cognitive worry items will yield stronger negative correlations, whereas measurement instruments dominated by physiological-affective symptom items will produce relatively weaker associations. This expectation is exploratory.

### 3.6 Formal research hypotheses

Drawing on social cognitive theory and control-value theory, the following hypotheses were formulated for the present meta-analysis:

*H1*. There will be a significant negative correlation between academic self-efficacy and test anxiety among Chinese secondary school students.

*H2*. Grade level will exert a significant moderating effect on the magnitude of the correlation between academic self-efficacy and test anxiety. The negative correlation will be stronger for senior high school students relative to junior high school students.

*H3*. Geographic region will exert a significant moderating effect on the magnitude of the correlation between academic self-efficacy and test anxiety. The negative correlation will be stronger for secondary school student samples from Eastern China compared with samples from Central and Western China.

*H4*. The type of academic self-efficacy measurement instrument will exert a significant moderating effect on the magnitude of the correlation between academic self-efficacy and test anxiety. Measurement instruments emphasising examination-relevant task content will yield stronger negative correlations with test anxiety, whereas measurement instruments focusing predominantly on general daily learning content will produce weaker negative associations.

*H5*. The type of test anxiety measurement instrument will exert a significant moderating effect on the magnitude of the correlation between academic self-efficacy and test anxiety. Measurement instruments emphasising cognitive worry items will yield stronger negative correlations, whereas measurement instruments dominated by physiological-affective symptom items will produce relatively weaker negative associations.

## 4 Materials and methods

### 4.1 Literature search and screening criteria

This study performed literature retrieval and screening in strict compliance with the PRISMA guidelines for systematic meta-analyses to enhance research transparency (Page et al., 2021). Chinese articles were collected from the CNKI database, while English articles were retrieved from Web of Science. Electronic database searches were formally conducted on May 1, 2026, covering publication dates from January 1, 2000, to April 30, 2026. For Chinese literature, searches were carried out using keyword combinations: academic self-efficacy, learning self-efficacy, exam anxiety, and test anxiety. The advanced retrieval formulas were set as “(academic self-efficacy + learning self-efficacy) * (test anxiety + exam anxiety)” and “self-efficacy * (test anxiety + exam anxiety)”, initially yielding 2,421 Chinese papers. Master’s and doctoral theses (grey literature sources) were retrieved from the dissertation sub-database within CNKI during the primary electronic search. While CNKI provides broad coverage of Chinese graduate theses, no additional independent thesis databases (e.g., Wanfang Data, CALIS) or specialised grey literature repositories were queried. For English language records, Web of Science built-in smart topic search was utilised with the keywords “Academic self-efficacy” and “Test anxiety”, generating 605 relevant articles. The complete Chinese and English search strings are provided in Supplementary materials. The initial total pool contained 3,026 records. Only Chinese and English language published records were eligible for screening. NoteExpress was used to remove duplicate records by checking record type, author, year, and title. A total of 251 duplicate records were removed, leaving 2,775 documents. Reference lists of all included primary studies and relevant published meta-analyses and systematic reviews were manually screened to capture potentially eligible records missed by database searches; no further eligible records were retrieved through this process.

Eligible records were required to recruit Chinese regular junior or senior high school participants, adopt clearly defined academic self-efficacy as the core construct, and report the complete zero-order correlation coefficient (*r*) between academic self-efficacy and test anxiety alongside the corresponding sample size (N) to enable meta-analytic effect-size pooling. Vocational and technical school students were excluded, given their distinct educational objectives, curricula, and evaluation systems compared with general-track secondary school students. Samples from disaster-stricken areas were also excluded, as traumatic contexts produce atypical self-cognition and anxiety responses that depart from ordinary school-based educational environments. Additional exclusion criteria included studies focusing solely on other self-efficacy variants (e.g., general self-efficacy, emotional regulatory self-efficacy, subject-specific self-efficacy) and non-empirical outputs (e.g., reviews, theoretical analyses, case studies).

Title-abstract screening and full-text eligibility assessment were completed independently by two researchers. A total of 104 documents (including theoretical studies, review articles, and conference abstracts) and 2,607 studies not addressing both academic self-efficacy and test anxiety were excluded, leaving 64 studies. Full-text review further excluded 29 studies with non-eligible samples (vocational/technical schools or disaster-stricken areas) and 5 studies lacking complete effect size (*r*) or sample size (N) data, leaving 30 studies ultimately included. All discrepancies were resolved through iterative open discussion between the two reviewers, and full consensus was reached with no unresolved disagreements. The literature search and screening process is illustrated in Figure 1.

**Figure 1:**
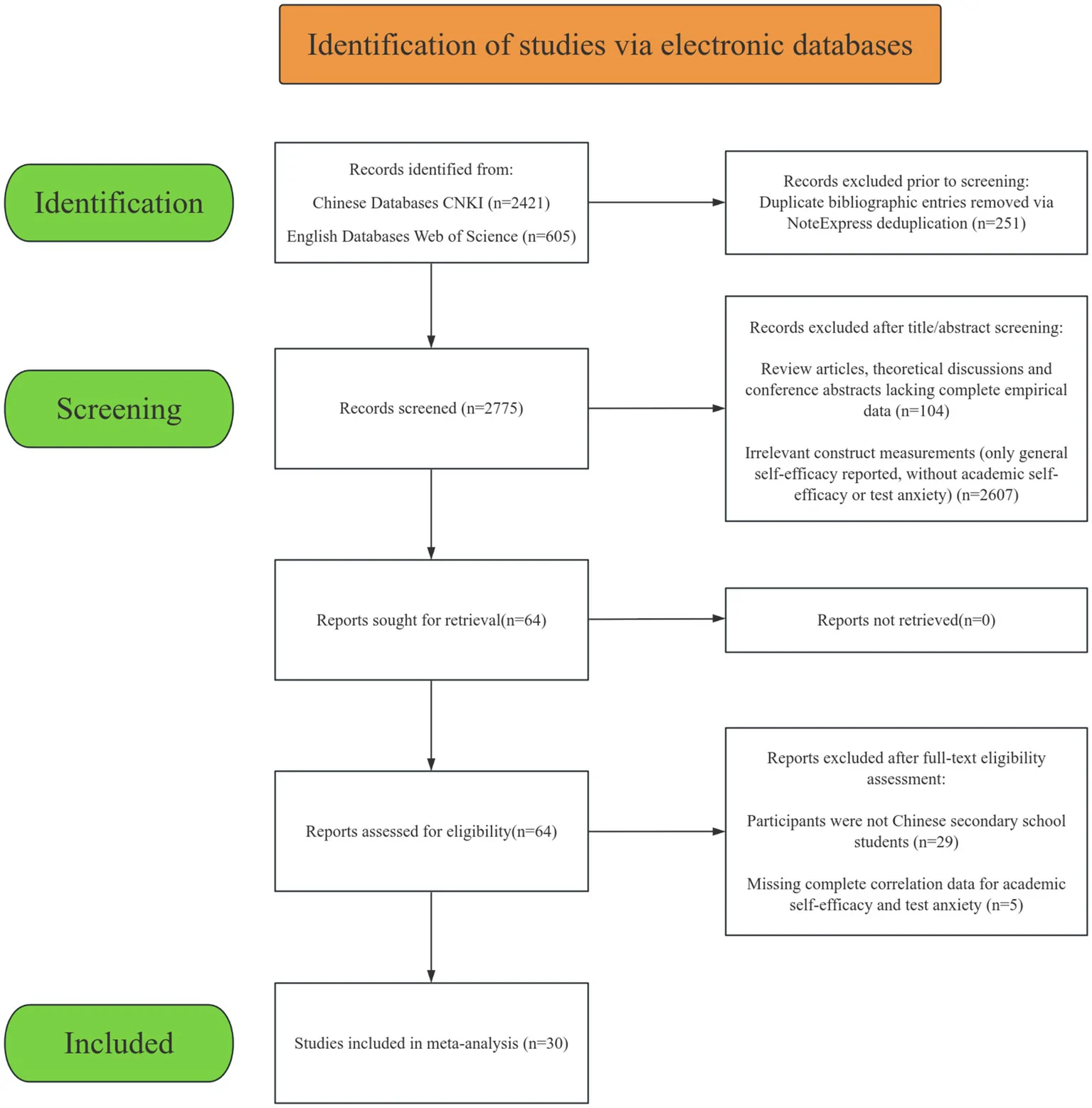
Literature screening process.

### 4.2 Literature quality assessment and coding

Two researchers independently extracted effect-size data and study characteristics from included articles. Discrepancies were resolved through group discussion by checking the original full-text publications. No additional data were requested from primary study authors, and no automated tools were applied for data extraction. Correlation coefficients between academic self-efficacy and test anxiety served as the primary outcome. To ensure the scientific soundness of the meta-analysis findings, the quality of the 30 finally included papers was assessed using the meta-analysis literature quality evaluation scale developed by Zhang et al. (2019). This criterion is based on items and standards for assessing experimental and intervention studies such as the Jadad scale, complying with international norms while remaining suitable for research conducted within the Chinese cultural context. The specific criteria are as follows: (1) Subject selection: 2 points for random sampling, 1 point for non-random sampling, and 0 points if no sampling method is reported. (2) Valid data rate: 2 points for a valid data rate of 0.9 and above, 1 point for a rate between 0.8 and 0.9, and 0 points for a rate below 0.8 or if the figure is unreported. (3) Internal consistency reliability of measurement instruments: 2 points for reliability coefficients of 0.8 and above, 1 point for coefficients ranging from 0.7 to 0.8, and 0 points for coefficients below 0.7 or if reliability data are not provided. (4) Journal tier: Journals indexed in CSSCI (including its extended version) and SSCI earn 2 points; Peking University Core Journals earn 1 point; general journals and unpublished papers earn 0 points. Total quality scores ranged from 0 to 8, with higher scores indicating better methodological quality. Scores of 0–3 represented low methodological quality, scores of 4–5 represented moderate methodological quality, and scores of 6–8 represented high methodological quality. All 30 papers scored 4 or higher, indicating satisfactory literature quality and no need for further exclusion.

The 30 studies were coded on the basis of authors, publication year, publication type, effect size (*r*), and sample size (N). The first author of each study was used as the coding reference. Publication types comprised five categories: Master’s and doctoral theses, SSCI journals, Core journals, General journals, and other types. Two researchers conducted the coding, and the Kappa coefficient for coding consistency was 0.907, indicating reliable agreement (Cohen, 1960). All discrepancies were resolved through iterative open discussion, and full consensus was reached with no unresolved disagreements. Table 1 provides an overview of the basic information of the included papers. Detailed descriptive information for each primary study, together with complete item-level quality-assessment scores, is provided in Supplementary materials.

**Table 1:** Basic characteristics of the literature included in the meta-analysis.

Author, Publication year	Publication type	*r*	n	Quality of literature	Academic self-efficacy measurement instrument	Test anxiety measurement instrument	Age range	Percentage of female (%)	Grade level	Geographic distribution
Chen and Ye (2007)	General journal	−0.400	3,511	4	Liang Yushong version scale	Tian Bao version scale	16–18 years old	47.3%	10–12 grades	Zhangzhou, Fujian province, China
Chen and Ye (2009)	Core journals	−0.470	799	5	Liang Yushong version scale	Tian Bao version scale	Not reported	50.1%	10 grade	Zhangzhou, Fujian province, China
Chen (2010)	General journal	−0.488	544	4	Liang Yushong version scale	Tian Bao version scale	16–18 years old	42.1%	10–12 grades	Zhangzhou, Fujian province, China
Cui (2022)	Master’s and doctoral Thesis	−0.329	952	6	Liang Yushong version scale	TAS	12–15 years old	Not reported	7–9 grades	Guilin City, Guangxi province (China)
Feng (2021)	Master’s and doctoral Thesis	−0.465	870	4	Liang Yushong version scale	TAI	16–18 years old	44%	10–12 grades	Nantong, Jiangsu province, China
Gu (2020)	Master’s and doctoral Thesis	−0.245	1,556	6	Liang Yushong version scale	TAS	14–18 years old	49.1%	10–12 grades	Changsha, Leiyang, Hunan province, China
Guo (2016)	Master’s and doctoral Thesis	−0.301	548	5	Liang Yushong version scale	TAS	16–18 years old	69.89%	10–12 grades	Baoding, Hebei province, China
Lei (2019)	General journal	−0.230	353	5	Liang Yushong version scale	TAS	16–18 years old	40.2%	10–12 grades	Chuzhou, Anhui province, China
Lei et al. (2021)	SSCI	−0.110	552	7	Liang Yushong version scale	TAS	15–18 years old	49.6%	10–12 grades	Shanxi province, China
Li (2020)	Master’s and doctoral Thesis	−0.244	6,203	6	Liang Yushong version scale	Li Jiahao version scale	12–14 years old	47%	7–8 grades	Henan province, China
Li (2022)	Master’s and doctoral Thesis	−0.450	655	5	Liang Yushong version scale	TAS	12–15 years old	49.5%	7–9 grades	Taiyuan, Shanxi province, China
Yang (2022)	Core journals	−0.270	1,013	7	Liang Yushong version scale	TAS	12–16 years old	56.6%	7–10 grades	Guizhou province and Chongqing municipality, China
Liu (2015)	Master’s and doctoral Thesis	−0.310	406	4	Liang Yushong version scale	TAI	12–15 years old	57.4%	7–9 grades	Hengyang, Hunan province, China
Liu (2012)	Master’s and doctoral Thesis	−0.429	302	5	Wang Zhenhong version scale	TAS	12–14 years old	51%	7–8 grades	Baicheng, Jilin province, China
Liu (2021)	Master’s and doctoral Thesis	−0.368	317	4	Liang Yushong version scale	TAS	Not reported	42.6%	9 grade	Kunming, Yunnan province, China
Ma and Li (2018)	General journal	−0.221	533	6	Liang Yushong version scale	Zheng Richang version scale	12–15 years old	48.8%	7–9 grades	Xingtai, Hebei province, China
Qin et al. (2017)	General journal	−0.364	214	4	Liang Yushong version scale	TAS	16–18 years old	55%	10–12 grades	Changzhi, Shanxi province, China
Qiu (2023)	Master’s and doctoral Thesis	−0.359	713	5	Liang Yushong version scale	TAS	12–15 years old	46.8%	7–9 grades	Nanning, Guangxi region, China
Shangguan and Du (2012)	General journal	−0.320	471	4	Hu Guiying version scale	TAS	16–17 years old	49.5%	10–11 grades	Jiangsu province, China
Tian et al. (2023)	General journal	−0.454	337	4	Liang Yushong version scale	TAS	16–18 years old	45.7%	10–12 grades	Wuhan, Hubei province, China
Wang and Huang (2014)	General journal	−0.340	297	6	Wang Zhenhong version scale	TAS	12–15 years old	50.5%	7–9 grades	Ningxia region, China
Xiang (2013)	Master’s and doctoral Thesis	−0.394	507	5	Liang Yushong version scale	TAS	16–18 years old	54.6%	10–12 grades	Changde, Hunan province, China
Xie (2013)	General journal	−0.792	442	4	Ge Yan version scale	Zheng Richang version scale	12–15 years old	43.9%	7–9 grades	Guangzhou, Guangdong province, China
Yang (2017)	Master’s and doctoral Thesis	−0.310	561	5	Liang Yushong version scale	TAS	Not reported	56.5%	12 grade	Luoping, Guizhou province, China
Yang (2010)	Master’s and doctoral Thesis	−0.636	297	4	Liang Yushong version scale,	TAS	16–18 years old	79.7%	10–12 grades	Hebei province, China
Zhao (2006)	Master’s and doctoral Thesis	−0.212	501	4	Zhao Jie version scale,	TAS	16–18 years old	53.3%	10–12 grades	Dingzhou, Hebei province, China
Zhao (2014)	Master’s and doctoral Thesis	−0.105	264	6	Liang Yushong version scale	Zheng Richang version scale	14–17 years old	43.9%	7–9 grades	Hohhot, Inner Mongolia region, China
Zhong (2021)	Master’s and doctoral Thesis	−0.340	3,263	4	Liang Yushong version scale	TAS	12–15 years old	45.2%	7–9 grades	Sichuan province, China
Zhou (2024)	Master’s and doctoral Thesis	−0.276	371	4	Liang Yushong version scale	TAS	12–14 years old	53.1%	7–8 grades	Beijing, China
Zhou (2019)	Master’s and doctoral Thesis	−0.227	513	6	Liang Yushong version scale	TAS	12–15 years old	49.3%	7–9 grades	Liaoning province, China

r, effect size. n, sample size.

Raw correlation coefficients *r* are bounded between −1 and 1 and follow a skewed distribution, violating the normality assumption required for meta-analytic pooling. All meta-analytic calculations were implemented via Comprehensive Meta-Analysis Version 3.0. In line with the built-in standardized workflow for correlation meta-analysis within CMA, every raw correlation coefficient was automatically transformed into Fisher’s Z scores before weighted pooling, following the formula:

$$Z_{r}=0.5\times \ln(\frac{1+r}{1-r})$$

Fisher’s Z values approximately conform to a normal distribution, enabling unbiased weighted effect-size synthesis under the random effects model. After CMA calculated the pooled Fisher’s Z value and its 95% confidence interval, the software automatically back-transformed the synthetic Z value to the original correlation coefficient *r*. When multiple correlation coefficients were reported within one study across distinct subgroups or measurement instruments, they were weighted by subgroup sample size and averaged to generate one effect size per study. When heterogeneous instruments were used to assess academic self-efficacy or test anxiety, only coefficients derived from validated measurement instruments targeting these two constructs were extracted; coefficients from irrelevant or off-target measurement instruments were excluded from effect-size pooling. None of the 30 included primary studies contained multiple eligible effect-size estimates, so this pre-specified averaging procedure was not applied. All 30 included studies reported negative correlation coefficients between academic self-efficacy and test anxiety, and all were retained in the present meta-analysis.

### 4.3 Coding of moderator variables

This study selected grade level, geographic region, academic self-efficacy measurement instruments and test anxiety measurement instruments as moderator variables for subgroup analysis. Since the subjects were middle and senior high school students, grade level was coded into two categories: junior high school and senior high school. Studies recruiting mixed samples of junior and senior high students without disaggregated effect-size estimates for each grade band were excluded from the grade-level moderation analysis. Only studies reporting pure junior high school samples (Grades 7–9) or pure senior high school samples (Grades 10–12) were retained. After exclusion, 29 studies entered this moderator analysis (14 junior high, 15 senior high).

Geographic region coding followed the official four-block economic-geographical classification from National Bureau of Statistics of China (2011), distinguishing Eastern, Central, Western, and Northeastern China. Although Northeastern China is an independent official region, it shares similar socioeconomic and educational resource characteristics with Eastern coastal areas. Given low statistical power from only two studies in the Northeastern subgroup, Northeastern samples were merged into the Eastern group for the primary analysis, forming three categories: Eastern (including Northeastern samples), Central, and Western. All 30 studies were included in this moderation analysis. For papers that did not explicitly specify the region, the default was the location of the author’s institution.

The coded academic self-efficacy measurement instruments included the Academic Self-Efficacy Questionnaire (ASEQ) developed by Wood, the Academic Self-Efficacy scale (ASE) developed by Chemers, the College Academic Self-Efficacy scale (CASES) developed by Owen, the Motivated Strategies for Learning Questionnaire (MSLQ) developed by Pintrich, the Morgan-Jinks Student Efficacy scale (MJSES) developed by Jinks, as well as scales independently compiled or revised by Chinese scholars. The specific categories of measurement instruments are presented in Table 2. Explicit item-level coding criteria were predefined prior to analyses. All academic self-efficacy instruments were assigned to three categories according to their original item content: the general learning-oriented category (measurement instruments predominantly assessing routine classroom learning with few examination-related items), the balanced category (measurement instruments giving comparable attention to everyday learning and exam-related efficacy beliefs), and the exam-oriented category (measurement instruments heavily centred on examination preparation and test-taking scenarios). This classification was based purely on item composition regardless of cultural origin. Within this framework, ASEQ, ASE, Bian Yufang version scale, Zeng Xinghua version scale, Liang Yushong version scale, Wang Zhenhong version scale, and Hu Guiying version scale were classified as balanced; CASES, MSLQ, MJSES, and Zhao Jie version scale were classified as general learning-oriented; and Ge Yan version scale was classified as exam-oriented.

**Table 2:** Detailed information of academic self-efficacy measurement instruments.

Instrument name	Author (compiler)	Detailed information	Applicable population
Academic Self-Efficacy Questionnaire (ASEQ)	Wood	There are 7 dimensions and a total of 29 original questions. The dimensions of this scale include: class concentration, memorization, exam concentration, understanding, explaining concepts, note-taking.	College students
Academic Self-Efficacy Scale (ASE)	Chemers	There are a total of 9 items, and they are scored using the Likert scale with 7 points. (1. I know how to schedule my time to accomplish my tasks. 2. I know how to take notes. 3. I know how to study to perform well on tests. 4. I am good at research and writing papers. 5. I am a very good student. 6. I usually do very well in school and at academic tasks. 7. I understand my academic tasks. 8. I find my university academic work is interesting. 9. I am very capable of succeeding at the university.)	College students
Bian Yufang version scale	Bian Yufang	This scale consists of 2 s-order dimensions (basic competence sense and control sense), and 10 first-order dimensions (belief in good learning outcomes, sense of goal achievement, talent sense, positive self-anticipation, self-confidence, self-doubt, susceptibility to interference, negative environmental perception, feeling of powerlessness, and effort sense). There are a total of 63 questions, using a 7-point Likert scoring system.	Secondary school students and primary school students
Ge Yan version scale	Ge Yan	Developed based on the Liang Yushong scale, this scale comprises two dimensions: self-efficacy for learning ability and self-efficacy for learning behavior. It contains 22 items rated on a 5-point Likert scale. All items are reworded to fit secondary school students, and its measurement content is highly oriented toward entrance examination preparation.	Secondary school students and college students
Hu Guiying version scale	Hu Guiying	This scale is divided into two dimensions: self-efficacy for learning behavior and self-efficacy for learning ability, containing 12 items rated on a 6-point Likert scale. It can measure individuals’ confidence in daily learning and exam performance.	Secondary school students
Liang Yushong version scale	Liang Yushong	This scale consists of two dimensions, namely self-efficacy for learning ability and self-efficacy for learning behavior, with a total of 22 items. It adopts a 5-point Likert scoring system and covers two domains: general daily learning and basic exam-related scenarios.	Secondary school students and college students
Morgan-Jinks Student Efficacy Scale (MJSES)	Jinks	There are three subscores: talent, context, effort. This scale consists of 30 questions and uses a 4-point Likert scoring system.	Children and teenagers
Motivated Strategies for Learning Questionnaire (MSLQ)	Pintrich	This scale is divided into 5 dimensions (Value component, Expectation component, Emotional component, Cognitive and metacognitive strategies, Resource management strategies), consisting of 50 questions in total.	College students
The College Academic Self-Efficacy Scale (CASES)	Owen	This scale consists of three dimensions: Overt, Social Situations, Cognitive Operations, Technical Skills. There are 33 questions, using the 5-point Likert confidence scoring system.	College students
Wang Zhenhong version scale	Wang Zhenhong	Based on Pintrich and De Groot’s MSLQ, there are a total of 24 questions, using a 6-point Likert scoring method.	Secondary school students
Zeng Xinghua version scale	Zeng Xinghua	Based on the Liang Yushong scale and the Bian Yufang scale, the scale consists of two dimensions: self-efficacy in learning ability and self-efficacy in learning behavior. It contains a total of 21 items and adopts the five-point Likert scoring method.	Secondary school students
Zhao Jie version scale	Zhao Jie	It was developed by the Insight Group of Peking University based on the academic efficacy component of the Adaptability Learning Model scale (PALS). It consists of 12 items to measure the self-efficacy in general learning abilities within the academic domain, using a five-point Likert scoring method.	Secondary school students

The coded test anxiety measurement instruments included Sarason’s Test Anxiety scale (TAS), Spielberger’s Test Anxiety Inventory (TAI), Cassady’s scale, as well as measurement instruments independently compiled or revised by Chinese scholars. Explicit item-level coding criteria were predefined prior to analyses. All test anxiety instruments were assigned to three categories according to their original item content documented in Table 3: the symptom-comprehensive oriented category (measurement instruments simultaneously assessing cognitive, physiological, and behavioural anxiety responses), the cognition-only oriented category (measurement instruments predominantly capturing cognitive domain anxiety without physical symptom items), and the affect-cognition oriented category (measurement instruments centred on emotional arousal and cognitive interference with few behavioural avoidance items). This classification was based purely on original scale item composition regardless of cultural origin. Within this framework, Sarason’s Test Anxiety scale (TAS) together with its Chinese Wang Caikang revision, Zheng Richang version scale, Tian Bao version scale, and Li Jiahao version scale were classified as symptom-comprehensive oriented; Cassady’s version scale was classified as cognition-only oriented; and Spielberger’s Test Anxiety Inventory (TAI) was classified as affect-cognition oriented.

**Table 3:** Detailed information of test anxiety measurement instruments.

Instrument name	Author (compiler)	Detailed information	Applicable population
Cassady version scale	Cassady	This scale is a single-dimensional scale consisting of 27 questions. It uses a 4-point Likert positive scoring system and is specifically designed to measure cognitive test anxiety throughout the entire process of exam preparation and taking the test, without including any items related to physical anxiety.	Secondary school students and college students
Li Jiahao version scale	Li Jiahao	This scale consists of 31 self-report items and adopts a 4-point Likert scoring method. It is composed of three dimensions: cognition, physiology and behavior. The cognitive dimension includes negative thoughts such as fear of poor grades, upward social comparison with peers, and rumination over potential bad consequences of examinations. The physiological dimension assesses somatic stress symptoms, for instance inner restlessness and rapid heartbeat during tests. The behavioral dimension captures anxiety-linked performance manifestations, including distracted attention and impaired cognitive processing while completing exam-related learning tasks.	Secondary school students
Test Anxiety Inventory (TAI)	Spielberger	This scale consists of 20 questions and uses a four-level Likert scoring method. It is divided into two dimensions: emotional response and cognitive interference. If the score is below 35, it indicates a low level of test anxiety. If the score is above 50, it indicates a high level of test anxiety.	Secondary school students and college students
Test Anxiety Scale (TAS)	Sarason	This scale consists of three dimensions: physical reaction, psychological reaction and behavioral reaction. It contains a total of 37 questions and is scored using a 1 and 0 system. A TAS score below 12 indicates mild anxiety, scores between 12 and 20 indicate moderate anxiety, and scores above 20 indicate severe anxiety.	Secondary school students and college students
Tian Bao version scale	Tian Bao	This scale consists of 28 self-report items and adopts a 5-point Likert rating system. It is composed of three dimensions: cognitive response, physiological arousal, and behavioral performance. Its items reflect test anxiety experiences of Chinese secondary school students, covering cognitive worries about failure and negative self-evaluation, physiological stress symptoms such as palpitations, trembling, and poor sleep quality, as well as behavioral-oriented responses including the urge to withdraw from examinations.	Secondary school students
Wang Caikang version scale	Wang Caikang and Liu Yong	A comprehensive translation of TAS has been carried out. The dimensions, the number of questions, and the scoring method are all the same as those of TAS.	Secondary school students and college students
Zheng Richang version scale	Zheng Richang	Composed of 33 self-report items, this scale adopts a 4-point Likert scoring system. Based on the total test anxiety score, participants can be classified into four severity levels in ascending order: calm, mild test anxiety, moderate test anxiety, and severe test anxiety. Its items cover somatic-physiological reactions (e.g., diarrhea, cold hands, headache, sweating), cognitive interference (e.g., difficulty concentrating, impaired memory retrieval, negative worry), as well as behavioural responses and subjective attitudes (e.g., aversion to examinations, examination-related dreams).	Secondary school students

For categorical moderator variables examined via both subgroup omnibus QBET tests and Knapp-Hartung corrected mixed-effects meta-regression, a robust moderating inference requires convergent statistical evidence from both analytical tools together with a positive analog *R*^2^. Subgroup results from categories containing only one or two primary studies are interpreted with caution even when statistical significance is observed. Missing information from primary studies was coded as missing without imputation.

### 4.4 Data analysis process

Fisher’s z transformation was used to convert correlation coefficients, and these converted values served as the main effect size for combining studies; all data analyses were carried out with Comprehensive Meta-Analysis V3.0 software. Correlation coefficients (*r*) and sample sizes (N) were extracted from eligible primary studies and entered according to standard meta-analysis data entry rules to ensure accuracy and consistency. The meta-analytic work included four parts; first, publication bias tests and sensitivity analyses were used to check possible publication bias and test whether the results were stable; second, how much the results varied across studies was tested, and this information was used to help choose the overall effect model; third, an overall main effect analysis was used to estimate the direction and size of the link between academic self-efficacy and test anxiety among secondary students; fourth, multi-dimensional moderation analysis was carried out for four categorical moderators through subgroup analysis, including grade level, geographic region, academic self-efficacy measurement instruments, and test anxiety measurement instruments. For categorical moderator tests, a strong moderator effect was defined in advance as meeting three conditions at the same time: a significant omnibus QBET from subgroup analysis, a significant omnibus *F*-test from Knapp-Hartung corrected meta-regression, and an analog *R*^2^ larger than zero, which would mean the moderator explained meaningful between-study variance. The Knapp-Hartung correction was applied to all mixed-effects meta-regression models; this correction changes the degrees of freedom and standard errors of regression coefficients, so it reduces inflated Type I error rates and gives more reliable *t*- and *F*-test results when the number of included studies is moderate. The analog *R*^2^ reported in this study measures how much of the between-study heterogeneity is explained by each meta-regression covariate; in Comprehensive Meta-Analysis V3.0, analog *R*^2^ is calculated as the proportional reduction in the estimated remaining between-study variance (*τ*^2^) after adding the predictor, compared with the baseline *τ*^2^ from the random effects model without covariates.

## 5 Results

The 30 included studies involved a total of 27,865 Chinese secondary school students. Participants comprised junior high school (Grades 7–9) and senior high school (Grades 10–12), with ages predominantly ranging from 12 to 18 years. Age-range information was not reported in a small subset of studies. The proportion of female participants varied across studies, and gender-ratio data were unreported for several samples. Study samples were distributed across multiple provincial-level regions of Eastern, Central, and Western China. Cronbach’s α reliability coefficients for measurement instruments were partially missing across primary studies. Other participant-level information, including socioeconomic status, was rarely documented in the included literature and could not be systematically coded.

### 5.1 Publication bias tests and heterogeneity tests

#### 5.1.1 Publication bias tests

Six complementary statistical methods were adopted to evaluate potential publication bias: funnel plot visual inspection, Egger’s linear regression test, Duval and Tweedie’s random-effects trim-and-fill correction, subgroup comparison of graduate theses versus journal articles, Classic fail-safe N analysis, and Begg and Mazumdar’s rank correlation test.

First, visual inspection of the funnel plot revealed mild asymmetry, suggesting a potential shortage of unpublished studies reporting weak or non-significant negative correlations on the left side of the mean effect size. The funnel plot for publication bias assessment is shown in Figure 2.

**Figure 2:**
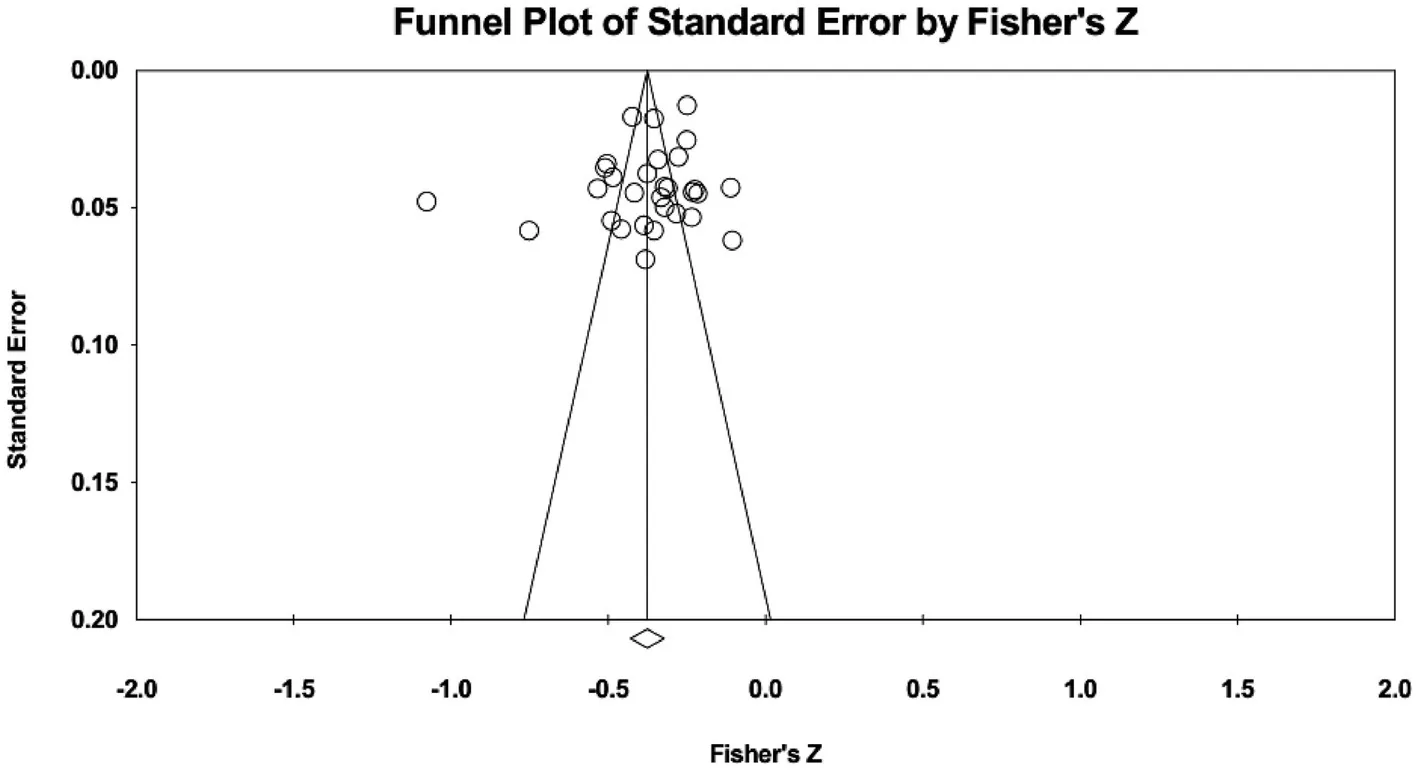
Publication bias detection funnel plot.

Second, Egger’s regression test was performed to quantify funnel asymmetry. The intercept was −2.282 (*SE* = 1.694), with a two-tailed 95% confidence interval of [−5.751, 1.188]. The two-tailed *p* = 0.189 > 0.05, indicating no statistically significant asymmetry at the conventional α = 0.05 threshold.

Third, Duval and Tweedie’s trim-and-fill analysis was run under the random-effects framework matching the primary model. Bilateral tests were added to verify the direction of asymmetry. Under the primary left-side missing assumption, 10 unreported studies were imputed to restore symmetry. The pooled correlation shifted from *r* = −0.359 to *r* = −0.426. This 18.7% increase in magnitude indicates the original estimate is a conservative approximation of the true effect. The negative association remained significant after correction, so the core conclusion of a significant moderate negative correlation remains unchanged. The right-side test imputed zero missing studies and returned an identical effect size, confirming asymmetry is concentrated on the left, consistent with standard publication bias patterns.

Fourth, subgroup analysis comparing effect sizes from graduate theses and journal articles was performed under the mixed-effects model to test journal-driven publication bias. The pooled correlation for graduate theses (k = 18) was *r* = −0.338, 95% CI [−0.382, −0.292], *p <* 0.001. For journal articles (k = 12), the pooled correlation was *r* = −0.390, 95% CI [−0.486, −0.286], *p <* 0.001. The between-group difference was non-significant (QBET = 0.852, *df* = 1, *p* = 0.356), indicating no statistically distinguishable difference in effect magnitude between graduate theses and journal articles within the present sample.

Fifth, Classic fail-safe N (Rosenthal, 1979) further verified the robustness of the significant pooled effect. Using a two-tailed significance criterion of *p* < 0.05, the analysis showed that at least 2,790 missing studies with zero correlation would be required to render the overall meta-analytic result non-significant (*p* > 0.05). This value vastly exceeded Rosenthal’s conventional critical benchmark of 5 k + 10 (5 × 30 + 10 = 160), providing strong evidence that unpublished null findings are unlikely to offset the current significant negative correlation.

Sixth, Begg and Mazumdar’s rank correlation test was conducted as a complementary assessment of publication bias. Under the random-effects framework, the test yielded Kendall’s *τ* = −0.048 (continuity-corrected), *p* = 0.708 (two-tailed). This result was consistent with Egger’s test in showing no statistically significant rank correlation between effect sizes and their standard errors; however, both tests have limited statistical power at k = 30, so a non-significant result does not rule out the subtle asymmetry detected by trim-and-fill.

Taken together, although trim-and-fill detected notable asymmetry and a non-trivial shift in effect magnitude, converging evidence confirms publication bias does not invalidate the core finding. The discrepancy between the non-significant Egger’s test (*p* = 0.189) and the ten studies imputed by trim-and-fill warrants interpretation. Egger’s regression test has limited statistical power when the number of primary studies is moderate (k = 30), and it may fail to detect subtle funnel asymmetry that trim-and-fill, a more sensitive data-imputation approach, can identify. Visual inspection of the funnel plot also revealed mild left-side asymmetry, consistent with the trim-and-fill finding rather than the null Egger’s result. Therefore, the non-significant Egger’s test should not be interpreted as evidence of complete absence of publication bias. The bias is meaningful but does not overturn the main results.

#### 5.1.2 Heterogeneity tests

Heterogeneity test results based on all 30 primary studies are presented in Table 4. Cochran’s *Q* = 546.689, *df* = 29, *p <* 0.001, and *I*^2^ = 94.695%. Following standard benchmarks, the present study exhibited extreme between-study heterogeneity. Such an extreme *I*^2^ value indicates that discrepancies in observed correlation coefficients were not merely generated by random sampling error; latent methodological and contextual moderators contribute substantial cross-study variance. To systematically unpack the sources of this severe heterogeneity, subgroup analyses and Knapp-Hartung corrected mixed-effects meta-regression were subsequently implemented.

**Table 4:** Results of heterogeneity test and overall pooled effect.

Model	k	Pooled *r*	95% CI lower	95% CI upper	Two-tailed test		Test for Heterogeneity		
					*Z*-value	*p*-value	*Q*	*df*	*I* ^2^
Fixed effects	30	−0.334	−0.345	−0.324	−57.933	0.000	546.689	29	94.695%
Random effects	30	−0.359	−0.405	−0.312	−13.722	0.000			

Given the extreme between-study heterogeneity (I^2^ = 94.695%), the random effects model was adopted as the primary analytical framework; fixed effects results are provided as a reference. Pooled r = pooled correlation coefficient. k = number of included studies. 95% CI lower and 95% CI upper = lower and upper bounds of the 95% confidence interval for pooled r. Z = test statistic for evaluating whether pooled r differs significantly from zero. p = corresponding p-value for the significance test of pooled r.

The 95% prediction interval (PI) for the overall random-effects correlation was [−0.579, −0.083], derived using the null-model between-study variance *τ*^2^ = 0.0205. Unlike the 95% confidence interval that only quantifies uncertainty around the average population correlation, the broad 95% prediction interval integrates both sampling error and cross-study heterogeneity, illustrating that the strength of the association between academic self-efficacy and test anxiety fluctuates substantially across distinct research conditions. Although the entire prediction interval remained negative, its upper bound (−0.083) was close to zero, indicating that the magnitude of the negative association could vary substantially across future studies. Specifically, some future studies with particular sample characteristics or measurement contexts might observe very small or even statistically non-significant negative correlations, whereas others could identify moderate to strong negative associations. This wide prediction interval is consistent with the extreme between-study heterogeneity (*I*^2^ = 94.695%) observed in the present dataset.

#### 5.1.3 Sensitivity analyses

Three complementary sensitivity checks were conducted to test the robustness of the overall correlational result: iterative leave-one-out exclusion, identification of influential studies, and outlier removal. All analyses adopted the random-effects framework matching the primary pooled model.

First, leave-one-out sensitivity analysis was performed to verify whether any single primary study could substantially distort the overall pooled correlation. After sequentially omitting each study and recalculating the weighted effect size, the pooled r values ranged from −0.367 to −0.338. Regardless of which single study was excluded, the corresponding 95% confidence interval consistently overlapped with the full-sample baseline interval [−0.405, −0.312]. This finding indicates that removing any individual study could not meaningfully shift the magnitude or statistical significance of the overall negative correlation.

Second, two studies (Xie, 2013; Zhao, 2014) were identified as producing the maximum fluctuation in pooled effect sizes during iterative exclusion. Specifically, Xie (2013) reported the strongest negative correlation (*r* = −0.792) among all included studies and was the only investigation adopting the Ge Yan version academic self-efficacy measurement instruments, with a sample from Guangzhou, Guangdong Province (Eastern China). In contrast, Zhao (2014) yielded the weakest negative correlation (*r* = −0.105) with a relatively small sample (*N* = 264) from Hohhot, Inner Mongolia (Western China), using the Liang Yushong version measurement instruments. These two studies occupied opposite ends of the effect-size distribution, which accounts for their disproportionate influence on the pooled estimate.

Third, the overall meta-analytic model was re-estimated after excluding these two influential studies, leaving 28 primary studies for re-pooling. The adjusted pooled *r* was −0.345, with a 95% CI of [−0.381, −0.307], *p <* 0.001. The revised confidence interval largely overlapped with the baseline full-sample interval, and the negative association remained highly statistically significant.

Collectively, all three sensitivity tests confirm that the observed negative correlation between academic self-efficacy and test anxiety is statistically robust, and the core finding is not driven by a small set of extreme outlier observations. Complete outputs of the leave-one-out analysis are provided in the Supplementary materials, and the results after removing influential studies are shown in Table 5.

**Table 5:** Results of outlier sensitivity analysis.

Effect Model	Pooled *r*	95% CI lower	95% CI upper	*Z*-value	*P*-value	*Q*-value	*df*	*I* ^2^
Fixed effect	−0.326	−0.336	−0.315	−55.638	0.000	295.503	27	90.863%
Random effects	−0.345	−0.381	−0.307	−16.769	0.000			

Results after removing Xie (2013) and Zhao (2014). Fixed effects results are provided as a reference. Pooled r = pooled correlation coefficient. 95% CI lower and 95% CI upper = lower and upper bounds of the 95% confidence interval for pooled r. Z = test statistic for evaluating whether pooled r differs significantly from zero. p = corresponding p-value for the significance test of pooled r.

### 5.2 Overall correlation between academic self-efficacy and test anxiety

Given the extreme between-study heterogeneity (*I*^2^ = 94.695%), a random effects model was selected to synthesize the average correlation magnitude. As shown in Table 4, the pooled correlation was *r* = −0.359, 95% CI [−0.405, −0.312], *Z* = -13.722, *p <* 0.001. According to Cohen’s standard for correlation effect sizes (|*r*| = 0.1 represents a small effect, |*r*| = 0.3 a moderate effect, and |*r*| = 0.5 a large effect) (Cohen, 2013), a statistically significant moderate negative correlation was observed between academic self-efficacy and test anxiety among Chinese secondary school students. All individual study effect sizes are visualized in the forest plot in Figure 3. The single pooled *r* only reflects the central tendency across heterogeneous samples. The wide 95% prediction interval [−0.579, −0.083] indicates considerable cross-sample fluctuation, so this averaged estimate cannot be generalized uniformly to all subgroups.

**Figure 3:**
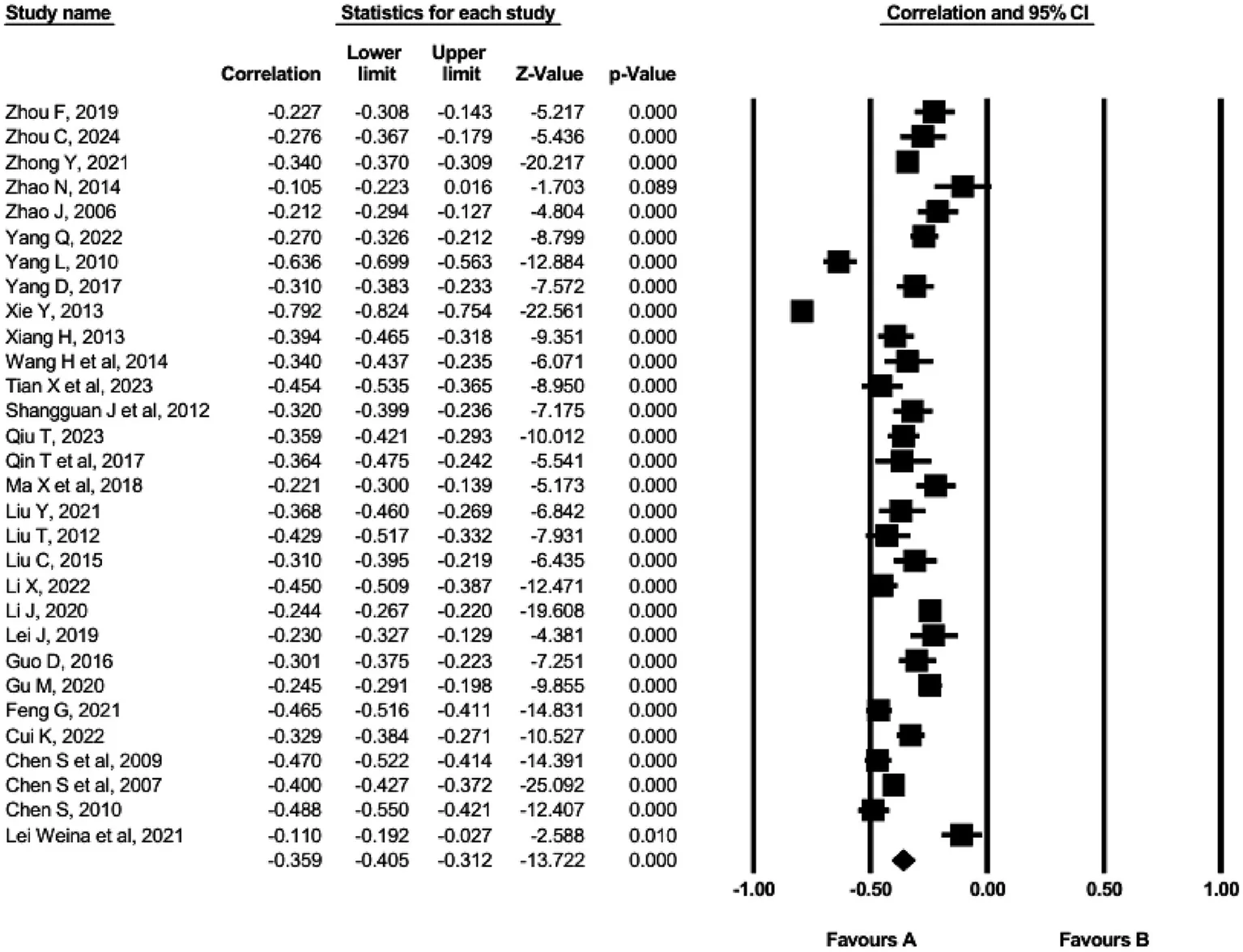
Forest plot.

### 5.3 Subgroup moderator analyses

Four categorical moderators were examined via mixed-effects subgroup analysis: grade level, geographic region, academic self-efficacy measurement instruments, and test anxiety measurement instruments. The omnibus between-group test (QBET) was used to examine overall differences across subgroups. Several subgroups contained only one or two primary studies. While the omnibus test based on the full sample supports general inferences, effect sizes from these small subgroups lack sufficient statistical power and should be interpreted with caution.

#### 5.3.1 Moderator: grade level

Subgroup results for junior high school (k = 14) and senior high school (k = 15) students were summarized in Table 6. Pooled *r* for junior high school = −0.358, 95% CI [−0.434, −0.277]; senior high school pooled *r* = −0.366, 95% CI [−0.425, −0.304]. QBET = 0.026, *p* = 0.871 > 0.05, demonstrating grade level exerted no significant moderating effect.

**Table 6:** Subgroup analysis: grade level.

Subgroup	k	Pooled *r*	95% CI lower	95% CI upper	*Z*	*P*	QBET	PBET
Junior high school	14	−0.358	−0.434	−0.277	−8.163	0.000	0.026	0.871
Senior high school	15	−0.366	−0.425	−0.304	−10.787	0.000		

Pooled r = pooled correlation coefficient. k = number of included studies. 95% CI lower and 95% CI upper = lower and upper bounds of the 95% confidence interval for pooled r. Z = test statistic for evaluating whether pooled r differs significantly from zero. p = corresponding p-value for the significance test of pooled r. QBET, between-group heterogeneity test statistic. PBET, *p*-value for QBET.

#### 5.3.2 Moderator: geographic region

Three regional subgroups were analyzed (Eastern k = 13, Central k = 9, Western k = 8). Pooled correlations ranged from −0.421 to −0.311; QBET = 4.721, *p* = 0.094 > 0.05, indicating region was not a significant moderator. The Western subgroup showed a notably narrower 95% CI [−0.351, −0.270] than the Eastern and Central subgroups, reflecting low within-group heterogeneity and a large aggregated sample size across its eight studies. This statistical feature does not alter the overall non-significant between-group result. The subgroup results for geographic region are summarized in Table 7.

**Table 7:** Subgroup analysis: geographic region.

Subgroup	k	Pooled *r*	95% CI lower	95% CI upper	*Z*	*P*	QBET	PBET
Eastern	13	−0.421	−0.506	−0.328	−8.127	0.000	4.721	0.094
Central	9	−0.312	−0.375	−0.245	−8.720	0.000		
Western	8	−0.311	−0.351	−0.270	−14.144	0.000		

Pooled r = pooled correlation coefficient. k = number of included studies. 95% CI lower and 95% CI upper = lower and upper bounds of the 95% confidence interval for pooled r. Z = test statistic for evaluating whether pooled r differs significantly from zero. p = corresponding *p*-value for the significance test of pooled r. QBET, between-group heterogeneity test statistic. PBET, *p*-value for QBET.

#### 5.3.3 Moderator: academic self-efficacy measurement instruments

Five academic self-efficacy measurement instruments subgroups were included: Liang Yushong version scale (k = 25), Wang Zhenhong version scale (k = 2), Hu Guiying version scale (k = 1), Ge Yan version scale (k = 1), Zhao Jie version scale (k = 1). All subgroups yielded significant negative correlations. Omnibus QBET = 221.754, *p <* 0.001, confirming measurement instrument type significantly moderated correlation magnitude. However, given that four of the five subgroups contained only one or two studies and the Ge Yan version subgroup (k = 1) reported an extreme effect size (*r* = −0.792), this omnibus result should be interpreted as preliminary evidence of moderation. The subgroup results for academic self-efficacy measurement instruments are summarized in Table 8.

**Table 8:** Subgroup analysis: academic self-efficacy measurement instruments.

Subgroup	k	Pooled *r*	95% CI Lower	95% CI Upper	*Z*	*P*	QBET	PBET
Liang Yushong version scale	25	−0.340	−0.380	−0.299	−15.121	0.000	221.754	0.000
Wang Zhenhong version scale	2	−0.386	−0.469	−0.295	−7.777	0.000		
Hu Guiying version scale	1	−0.320	−0.399	−0.236	−7.175	0.000		
Ge Yan version scale	1	−0.792	−0.824	−0.754	−22.561	0.000		
Zhao Jie version scale	1	−0.212	−0.294	−0.127	−4.804	0.000		

Pooled r = pooled correlation coefficient. k = number of included studies. 95% CI lower and 95% CI upper = lower and upper bounds of the 95% confidence interval for pooled r. Z = test statistic for evaluating whether pooled r differs significantly from zero. p = corresponding *p*-value for the significance test of pooled r. QBET, between-group heterogeneity test statistic. PBET, *p*-value for QBET. Subgroups with k ≤ 2 have low statistical power, and their results should be interpreted with caution.

#### 5.3.4 Moderator: test anxiety measurement instruments

Five test anxiety measurement instruments subgroups: TAS (k = 21), TAI (k = 2), Zheng Richang version scale (k = 3), Tian Bao version scale (k = 3), Li Jiahao version scale (k = 1). QBET = 43.549, *p <* 0.001, indicating significant moderation by test anxiety measurement instruments. The subgroup results for test anxiety measurement instruments are summarized in Table 9.

**Table 9:** Subgroup analysis: test anxiety measurement instruments.

Subgroup	k	Pooled *r*	95% CI lower	95% CI upper	*Z*	*P*	QBET	PBET
TAS	21	−0.333	−0.374	−0.291	−14.619	0.000	43.549	0.000
TAI	2	−0.393	−0.534	−0.232	−4.544	0.000		
Zheng Richang version scale	3	−0.438	−0.792	0.136	−1.517	0.129		
Tian Bao version scale	3	−0.448	−0.506	−0.386	−12.614	0.000		
Li Jiahao version scale	1	−0.244	−0.267	−0.220	−19.608	0.000		

Pooled r = pooled correlation coefficient. k = number of included studies. 95% CI lower and 95% CI upper = lower and upper bounds of the 95% confidence interval for pooled r. Z = test statistic for evaluating whether pooled r differs significantly from zero. p = corresponding *p*-value for the significance test of pooled r. QBET, between-group heterogeneity test statistic. PBET, *p*-value for QBET. Subgroups with k ≤ 2 have low statistical power, and their results should be interpreted with caution.

### 5.4 Mixed-effects meta-regression analysis

To further decompose the extreme cross-study heterogeneity observed in the overall dataset, three separate mixed-effects meta-regression models with REML estimation and Knapp-Hartung correction were constructed. One model treated study-level sample size as a continuous covariate. The other two models adopted dummy-coding for categorical predictors, representing academic self-efficacy measurement instruments and test anxiety measurement instruments, respectively. These meta-regression analyses supplemented subgroup analyses: the continuous model examined whether sample size accounted for cross-study variance, while the dummy-coded models quantified the proportion of between-study variance explained by measurement instrument characteristics.

#### 5.4.1 Meta-regression: sample size

To test whether study-level sample size predicted variation in effect-size estimates, a separate Knapp-Hartung corrected mixed-effects meta-regression was fitted with sample size entered as a continuous covariate. Meta-regression indicated that primary-study sample size was not a significant predictor of effect size, *β* = 0.0000, 95% CI [−0.0000, 0.0001], *t*(28) = 0.66, *p* = 0.5172. The analog *R*^2^ suggested that sample size explained almost no between-study heterogeneity. Note that meta-regression analyses in the present study are exploratory and should be interpreted with caution.

#### 5.4.2 Meta-regression: academic self-efficacy measurement instruments

The widely used Liang Yushong version scale was set as the reference group. The omnibus *F*-test was significant (*F* = 6.85, *df*_1_ = 4, *df*_2_ = 25, *p* = 0.0007), indicating that the set of measurement instruments variables as a whole significantly predicted correlation differences. Null model *τ*^2^ = 0.0205; residual *τ*^2^ after adjustment = 0.0118, analog *R*^2^ = 0.43. This indicates that 43% of raw between-study random variance could be explained by differences in academic self-efficacy measurement instruments. Residual *I*^2^ remained 91.65%, *Q* = 299.25, *p <* 0.001, indicating that substantial unexplained heterogeneity remained after controlling for measurement instruments type. Both tests reached significance, but post-hoc contrasts showed that only the single-study Ge Yan subgroup (Xie, 2013, *r* = −0.792) differed from the reference group (*b* = −0.7226, *p* < 0.001), with all other comparisons non-significant. The overall moderating effect is thus largely driven by this one outlying study and should be treated as preliminary suggestive evidence pending replication.

#### 5.4.3 Meta-regression: test anxiety measurement instruments

Subgroup analysis revealed a statistically significant overall between-group difference across test anxiety measurement instruments (QBET = 43.549, *p <* 0.001). However, categorical meta-regression provided consistent evidence that test anxiety scale type did not exert a meaningful moderating effect. With the TAS scale set as the reference group, the omnibus *F*-test for the full set of scale categories was non-significant (*F* = 0.72, *df*_1_ = 4, *df*_2_ = 25, *p* = 0.589), indicating that test anxiety measurement instruments failed to predict cross-study variation in effect sizes. The analog *R*^2^ equaled −0.09, indicating zero explanatory capacity for residual heterogeneity, and residual between-study heterogeneity remained extremely high (*I*^2^ = 93.60%, *p* = 0.001). Furthermore, pairwise comparisons between each alternative scale and the TAS reference group yielded no statistically significant differences (all *p* > 0.05). Notably, several subgroups contained a very limited number of primary studies (TAI: k = 2; Li Jiahao version scale: k = 1), which increased random sampling fluctuation and inflated the Type I error risk of the omnibus QBET test. Taken together, these results indicate that test anxiety measurement instruments do not significantly moderate the correlation between academic self-efficacy and test anxiety.

## 6 Discussion

### 6.1 Overall association between academic self-efficacy and test anxiety

H1 was supported. The combined effect size for the link between academic self-efficacy and test anxiety among Chinese secondary school students was *r* = −0.359, and according to Cohen’s guideline for correlation coefficients, this value shows a moderate negative association; in other words, students with higher academic self-efficacy tend to have lower test anxiety; this correlation explains about 13% of the differences in test anxiety, so academic self-efficacy can be seen as a practically meaningful factor related to test anxiety in Chinese secondary school settings; however, substantial residual heterogeneity remains, indicating that test anxiety among Chinese secondary school students is not solely moderated by academic self-efficacy; other potential moderators include parental rearing styles (Journault et al., 2025), life attitudes (Gajdošová et al., 2024), and related factors; it should be noted that this meta-analysis only combined correlational evidence from cross-sectional studies, so a moderate correlation cannot confirm a causal relationship.

### 6.2 Interpretation of non-significant moderators

#### 6.2.1 Grade level

H2 was not supported. Subgroup analyses showed that grade level did not have a significant moderating influence on the correlation between academic self-efficacy and test anxiety; although the combined correlation was slightly stronger among senior high school students than among junior high school students, this small difference did not reach statistical significance; according to control-value theory, test anxiety comes from two main cognitive judgments: one is how much control students feel they have over academic tasks, and the other is how important they think exam results are (Pekrun, 2006); in China’s unified exam system that is oriented toward sorting students into different school tracks, both junior and senior high school students are constantly exposed to pressure from being evaluated, junior high school students face the senior high school entrance exam streaming, while senior high school students face the college entrance exam pressure (Bian et al., 2025; Wu et al., 2025; Ren and Shen, 2025); family investment in extra tutoring keeps increasing, which also raises academic stress for adolescents (Ren and Liang, 2024); although the real-life results of exam failure are somewhat different across grade groups, students at both stages attach high importance to exam performance and see poor test results as a threat to academic development; this creates a shared thinking path that connects low feelings of control to test anxiety responses; even though absolute test anxiety scores tend to rise with grade advancement (Janjua et al., 2022), the strength of the correlation between academic self-efficacy and test anxiety stays stable across secondary school grades, which explains why grade level did not show a significant moderating influence. In addition, there may be a ceiling effect in test anxiety scores, and this can also make the correlation sizes similar across grade groups; if students at both stages already experience relatively high test anxiety under the unified high-stakes exam system, the limited range of anxiety scores could weaken the association between academic self-efficacy and test anxiety, making grade-level differences harder to detect.

#### 6.2.2 Geographic region

H3 was not supported. Subgroup analyses indicated that geographic region exerted no significant moderating effect on the correlation between academic self-efficacy and test anxiety. Although the pooled correlation for secondary school students from Eastern China presented a slightly stronger negative magnitude relative to students from other regions, this minor difference did not reach statistical significance. Despite marked disparities in educational resources across Eastern, Central, and Western China (Hao, 2021), students nationwide operate under identical national examination-driven evaluation mechanisms, including monthly assessments and academic rankings (Bian et al., 2025; Ren and Shen, 2025). Within this unified institutional context, the psychological process through which low perceived academic control triggers test anxiety exhibits little variation across regions (Pekrun, 2006), which explains the absence of geographic differences. Notably, within-region urban–rural disparities in educational resources may also exist, but these are similarly overridden by the uniform evaluation mechanism described above.

#### 6.2.3 Test anxiety measurement instruments

H5 was not supported. Although the omnibus QBET statistic from subgroup analysis reached significance (QBET = 43.549, *p <* 0.001), the Knapp-Hartung corrected meta-regression was non-significant (*F* = 0.72, *df*_1_ = 4, *df*_2_ = 25, *p* = 0.589) with a negative analog *R*^2^ (−0.09). Because this moderator failed to satisfy the three concurrent criteria for a robust effect specified *a priori* in Section 4.4, H5 was not supported. From control-value theory, test anxiety originates from low perceived control over academic performance and high subjective value placed on examination outcomes (Pekrun, 2006). The studies included in this meta-analysis adopted diverse test anxiety measurement instruments that varied in item structure, scoring rules, and dimensional emphasis. Some measurement instruments prioritize cognitive worry components, whereas others focus more heavily on physiological arousal and behavioral symptoms. Despite these structural differences, all instruments capture core test anxiety responses derived from the dual cognitive appraisal process defined by control-value theory.

Such consistency in underlying psychological mechanisms explains why instrument dimensional weighting failed to moderate the association between academic self-efficacy and test anxiety. The significant subgroup difference observed in the present study is most likely attributable to methodological limitations, including small sample sizes in certain subgroups and limited statistical power of categorical meta-regression, rather than genuine population level moderation effects.

### 6.3 Interpretation of significant moderators: academic self-efficacy measurement instruments

H4 receives only preliminary suggestive evidence. Academic self-efficacy measurement instrument type significantly moderated the correlation between academic self-efficacy and test anxiety.

All included academic self-efficacy measurement instruments were divided into three categories based on item content orientation: the general learning-oriented category, the balanced category, and the exam-oriented category.

The general learning-oriented category focuses purely on daily learning competence without exam-targeted items. The Zhao Jie version scale belongs to this category. It exclusively assesses general learning self-efficacy and generates the weakest negative correlation between academic self-efficacy and test anxiety among all subgroups. The balanced category covers both routine daily learning and basic exam-related perceptions, serving as the primary data source for the overall pooled effect size. Representative measurement instruments in this category include the Liang Yushong version scale, the Hu Guiying version scale, and the Wang Zhenhong version scale. As a classic balanced measurement instrument, the Liang Yushong version scale evaluates learning ability and learning behavior self-efficacy, covering both routine academic learning and basic exam scenarios. The Hu Guiying version scale retains general learning dimensions and basic pre-exam belief measurement but lacks items measuring long-term exam preparation perceived control. Revised from the Motivated Strategies for Learning Questionnaire, the Wang Zhenhong version scale adds metacognition and resource management components and further optimizes the measurement of exam preparation perceived control within a balanced theoretical framework.

The exam-oriented category emphasizes high-stakes entrance examination scenarios and prioritizes the measurement of exam-specific self-efficacy. The Ge Yan version scale, a revised version of the Liang Yushong version scale tailored for secondary school students, falls into this category. With enhanced item weighting on high-stakes entrance exam contexts, its comprehensive exam-specific content conforms to the dual appraisal mechanism of control-value theory in China’s competitive educational system. Accordingly, this scale yields the strongest negative correlation between academic self-efficacy and test anxiety across all included instruments.

Notably, several subgroups in the present categorical moderation analysis contain a relatively small number of primary studies. Accordingly, the claim that instrument type moderates the association should be regarded as tentative and in need of replication.

### 6.4 Unexplained residual sources of heterogeneity

Even after accounting for academic self-efficacy measurement instruments, considerable residual between-study heterogeneity remains. Drawing on social cognitive theory and control-value theory, several uncoded contextual factors could theoretically moderate the magnitude of the association between academic self-efficacy and test anxiety.

First, participant socioeconomic status shapes students’ access to resources and perceived academic pressure. Differences in academic self-efficacy beliefs and anxiety responses across family backgrounds may in turn alter this association.

Second, school type creates distinct academic environments. Intense peer competition in key-point secondary schools may modify the correlation between academic self-efficacy and test anxiety.

Third, urban–rural school background is associated with disparities in educational resources and social expectations, which may alter this association.

Fourth, the timing of questionnaire administration relative to high-stakes examinations plays an important role. Surveys conducted shortly before major examinations raise overall test anxiety levels, which may moderate the correlation between academic self-efficacy and test anxiety.

## 7 Conclusion

This meta-analysis finds a significant moderate negative correlation between academic self-efficacy and test anxiety among Chinese secondary school students (*r* = −0.359, 95% CI [−0.405, −0.312]).

Grade level, geographic region, and test anxiety measurement instruments do not significantly moderate the association, whereas academic self-efficacy measurement instruments do; instruments with more exam-oriented items yield stronger negative correlations. Nevertheless, given the limited number of studies in certain subgroups, this finding should be interpreted as preliminary suggestive evidence only. An exploratory analysis further indicates that study-level sample size does not predict effect size variation.

Grounded in social cognitive theory and control-value theory, these findings suggest that China’s unified high-stakes examination system creates comparable evaluative pressure across grades and regions, while variation in exam-oriented item coverage across academic self-efficacy measurement instruments alters the observed association magnitude.

## 8 Research contributions

### 8.1 Theoretical contribution

Prior empirical studies have consistently identified a negative correlation between academic self-efficacy and test anxiety among Chinese adolescents (Lei et al., 2021). However, isolated individual study findings cannot clarify whether the magnitude of this correlation varies as a function of grade level, geographic region, and measurement instruments. Existing meta-analyses reveal meaningful research gaps that the present study addresses. Most earlier syntheses have centred on general self-efficacy (Zhu, 2015); alternatively, some have focused on academic achievement or broad academic emotions rather than test anxiety specifically (Ji and Zhao, 2021). Compared with domain-specific academic self-efficacy, general self-efficacy yields weaker predictive power for test anxiety symptoms. Consequently, the distinct relationship between academic self-efficacy and test anxiety remains under-examined for Chinese secondary school student samples.

This study constitutes the first large-sample meta-analysis to systematically investigate the association between academic self-efficacy and test anxiety among Chinese secondary school students. It provides synthesized empirical evidence for research on adolescent academic emotions and further enriches empirical validation of social cognitive theory and control-value theory within the Chinese secondary school academic-affect context. This work also bears meaningful theoretical and practical implications for educational psychology.

### 8.2 Practical contributions

#### 8.2.1 Suggestions for primary and secondary school teachers

The present meta-analysis found a significant moderate negative correlation between academic self-efficacy and test anxiety. For teachers, this result suggests that students’ academic self-efficacy is associated with their test anxiety levels, and understanding this relationship may help teachers interpret students’ anxiety responses. Furthermore, academic self-efficacy measurement instrument type showed a statistically significant moderating association, although this finding is preliminary. With this caveat in mind, measurement instruments covering examination contexts may more sensitively capture the link between academic self-efficacy and test anxiety, and teachers may consider such instruments when evaluating students, though further validation is needed.

#### 8.2.2 Suggestions for school counselors

The present meta-analysis found that academic self-efficacy scale type significantly moderated the strength of the correlation between academic self-efficacy and test anxiety. For school counselors, this finding has direct implications for assessment practice. When conducting pre-test and post-test evaluations of counseling programs, measurement instruments covering both daily learning and exam scenarios should be prioritized, because general-learning-only measurement instruments tend to yield weaker associations and may underestimate the link between self-efficacy and test anxiety, potentially distorting judgments of intervention effects. Standardized assessment using appropriate measurement instruments therefore contributes to more accurate evaluation of counseling outcomes. However, given the preliminary nature of this moderator finding, counselors should not rely on instrument selection alone to judge intervention effects, and standardized assessment should be complemented by multiple sources of evidence.

#### 8.2.3 Suggestions for educational policy makers

The present meta-analysis found that the negative association between academic self-efficacy and test anxiety was stable across grade levels and geographic regions. For educational policy makers, this suggests that assessment and screening frameworks may be designed with broad applicability across secondary school grades and regions, as the strength of the association does not vary systematically by these demographic factors. However, this correlational finding does not imply uniform intervention effectiveness across all contexts; local evaluation remains necessary. In addition, academic self-efficacy measurement instrument type significantly moderated the correlation. Policy makers may consider the potential value of standardized academic self-efficacy measurement instruments covering both daily learning and exam scenarios for school-based mental health screening, but this recommendation should be regarded as exploratory pending further research with more diverse instrument types and larger independent samples.

## 9 Limitations and future research directions

### 9.1 Research limitations

First, several subgroups contained only one or two primary studies, reducing statistical power for both subgroup analysis and meta-regression. Notably, the Ge Yan version scale subgroup comprised only one study (Xie, 2013), making its meta-regression coefficient (*b* = −0.7226) and the corresponding analog *R*^2^ of 0.43 potentially unstable. Although convergent evidence from subgroup omnibus tests and Knapp-Hartung corrected meta-regression helps screen for spurious moderation signals, this approach cannot fully quantify Type I error rates for subgroup-level tests, and formal simulation analyses cannot be reliably parameterized with the current dataset. Inferences from single-study subgroups therefore remain tentative and require replication.

Second, this meta-analysis was not pre-registered on a professional platform. To ensure transparency, the complete retrieval strategy, database range, and time window (January 1, 2000 to April 30, 2026) are fully reported in the methodology section to facilitate replication.

Third, this meta-analysis synthesizes cross-sectional bivariate correlations only and therefore cannot support causal conclusions or examine multivariate relationships involving additional variables such as academic buoyancy or peer support. While low perceived control may trigger test anxiety, sustained high anxiety may also erode self-efficacy over time through persistent worry, cognitive interference during examinations, and repeated academic failure. Longitudinal designs, randomized controlled trials, or cross-lagged panel models are needed to disentangle the bidirectional causal relationship.

Fourth, publication bias evaluation has contextual and methodological limitations. Most included studies were retrieved from Chinese-language databases (primarily CNKI), which may not cover all regional journals and could omit some unpublished or non-indexed Chinese research; bias patterns in Chinese literature may also differ from those in Western English-language literature. Although a substantial number of graduate theses were included to mitigate journal-related publication bias, these evaluations cannot be directly generalized to non-Chinese research contexts. Additionally, selection-model-based adjustment was not performed because such models require extra parameters and yield unstable outputs with k = 30; multiple complementary detection methods were therefore used for triangulation.

Fifth, all samples are Chinese secondary school students embedded in a national high-stakes examination system with collectivist cultural values emphasizing academic performance. Given cross-cultural differences in assessment structures, social expectations, and the cultural significance of examination success, the findings cannot be directly generalized to other age groups, educational contexts, or cultural settings.

Sixth, the meta-analysis relied on study-level aggregated data, preventing examination of individual characteristics such as gender and socioeconomic status as moderators. Additionally, both academic self-efficacy and test anxiety were assessed via self-report measures, which may introduce common method variance and inflate observed correlations.

### 9.2 Future research directions

Future meta-analyses should incorporate more potential moderators to further unpack the sources of heterogeneity in the association between academic self-efficacy and test anxiety. In addition, longitudinal studies can be incorporated to further explore the bidirectional relationship between academic self-efficacy and test anxiety. Future meta-analyses may also employ Bayesian meta-regression to compute Bayes factors, which can formally quantify the relative strength of evidence for null versus alternative moderation effects, complementing the frequentist non-significant results obtained in the present study.

## Data Availability

The datasets presented in this study can be found in the Mendeley database (doi: 10.17632/xt5j2yjtkm.1) and in the Supplementary material of this article.

## References

[ref1] Bandura A. (1978). Self-efficacy: toward a unifying theory of behavioral change. Adv. Behav. Res. Ther. 1, 139–161. doi: 10.1016/0146-6402(78)90002-4 847061

[ref2] Bian X. Shen X. Weng L. (2025). From relaxing the Gaokao to inclusion in the Gaokao: institutional origins and reform evolution of the high school academic proficiency examination. Global Educ. 54, 73–91.

[ref3] Bie F. Yan X. Xing J. Wang L. Xu Y. Wang G. . (2024). Rising global burden of anxiety disorders among adolescents and young adults: trends, risk factors, and the impact of socioeconomic disparities and COVID-19 from 1990 to 2021. Front. Psych. 15:1489427. doi: 10.3389/fpsyt.2024.1489427, 39691785 PMC11651023

[ref4] Bol T. Van de Werfhorst H. G. (2013). Educational systems and the trade-off between labor market allocation and equality of educational opportunity. Comp. Educ. Rev. 57, 285–308. doi: 10.1086/669122

[ref5] Brandmo C. Braten I. Schewe O. (2018). Social and personal predictors of test anxiety among Norwegian secondary and postsecondary students. Soc. Psychol. Educ. 22, 43–61. doi: 10.1007/s11218-018-9461-y, 30311153

[ref6] Chen S. (2010). Relationship among behavior type, academic self-efficacy and test anxiety of senior high school students. China J. Health Psychol. 18, 70–73. doi: 10.13342/j.cnki.cjhp.2010.01.039

[ref7] Chen S. Ye G. (2007). Test anxiety of senior high school students and its relationship with academic self-efficacy. J. Minnan Normal Univ. 3, 133–137. doi: 10.16007/j.cnki.issn2095-7122.2007.03.029

[ref8] Chen S. Ye G. (2009). The relationship of test anxiety and academic achievement: the role of moderated mediator. Psychol. Res. 2, 74–79.

[ref9] Cohen J. (1960). A coefficient of agreement for nominal scales. Educ. Psychol. Meas. 20, 37–46. doi: 10.1177/001316446002000104

[ref10] Cohen J. (2013). Statistical Power Analysis for the Behavioral Sciences. New York, NY, USA: Routledge.

[ref11] Cui K. (2022). The Effect of Academic Self-Efficacy on Test Anxiety Among Secondary School Students: The Roles of Fear of Negative Evaluation and Core Self-Evaluations. (master’s thesis). Nanning, China: Guangxi Minzu University.

[ref12] Du X. Jia J. Duan T. Tao X. Li J. Yalikun S. . (2025). Trend of chinese adolescent anxiety symptoms before and after COVID-19: a cross-temporal meta-analysis with segmented regression. J. Res. Adolesc. 35:e70046. doi: 10.1111/jora.70046, 40671275

[ref13] Duan H. Yuan Y. Yang C. Zhang L. Zhang K. Wu J. (2015). Anticipatory processes under academic stress: an ERP study. Brain Cogn. 94, 60–67. doi: 10.1016/j.bandc.2015.01.002, 25659936

[ref14] Feng G. (2021). The Relationship Between Psychological Flexibility and Test Anxiety of Senior High School Students. (master’s thesis). Yangzhou University.

[ref15] Gajdošová M. Haláková Z. Adámková R. Kubiatko M. (2024). Exam anxiety in connection with life orientation in upper secondary education students. J. Child Adolesc. Ment. Health 36, 169–181. doi: 10.2989/17280583.2024.2393839, 39558638

[ref16] Gu M. (2020). The Influence of Parenting style of Senior High School Students on Test Anxiety: The Mediating Effect of Learning Self-Efficacy. (master’s thesis). Changsha, China: Hunan Agricultural University.

[ref17] Guo D. (2016). A Research on the Relationship Among Academic Self-Efficacy, Text Anxiety and Learning Burnout of the High School Students (master’s thesis). Hebei University.

[ref18] Hao W. (2021). Further intensifying efforts to narrow the education modernization gap between the east and the west and between urban and rural areas. J. Northwest Normal Univ. 58, 29–36. doi: 10.16783/j.cnki.nwnus.2021.06.004

[ref19] Huang Q. Zhou R. (2019). The development of test anxiety in Chinese students. Chin. J. Clin. Psychol. 27, 113–118. doi: 10.16128/j.cnki.1005-3611.2019.01.023

[ref20] Janjua Q. M. Khan F. Mukhtar S. (2022). Examination anxiety in integrated modular curriculum: perceptions of dental students. Health Prof. Educ. J. 5, 19–25. doi: 10.53708/hpej.v5i1.1265

[ref21] Ji C. Zhao H. (2021). The relationship between teacher support, academic self-efficacy, and academic achievement of primary and middle school students: a meta-analytic structural equation model. Teac. Educ. Res. 33, 106–113. doi: 10.13445/j.cnki.t.e.r.2021.06.008

[ref22] Journault A. Longpré C. Mageau G. A. Lupien S. J. (2025). Parental threat perception and hyper-parenting as potential risk factors for adolescents’ test anxiety. Soc. Dev. 34:1–15. doi: 10.1111/sode.70004

[ref23] Lei J. (2019). A correlational study on academic self-efficacy and test anxiety among senior high school students. Mental Health Educ. Primary Sec. Sch. 32, 21–23.

[ref24] Lei W. Zhang H. Deng W. Wang H. Shao F. Hu W. (2021). Academic self-efficacy and test anxiety in high school students: a conditional process model of academic buoyancy and peer support. Sch. Psychol. Int. 42, 616–637. doi: 10.1177/01430343211039265

[ref25] Li J. (2020). The Relationship Between Junior Middle School Student’s Test Anxiety and Academic Achievement: The Mediating Effect of Academic Self-Efficacy. (master’s thesis). Shanghai, China: East China Normal University.

[ref26] Li X. (2022). The Influence of Parental Psychological Control on Testanxiety of Junior High School Students: The Chain Mediating Effect of Self-Esteem and Academic Self-Efficacy. (master’s thesis). Taiyuan, China: Shanxi University.

[ref27] Liu T. (2012). A Study on the Relationship of Academic Adaptability, Test Anxiety, Academic Self-Efficacy and Academic Achievement Among Junior Middle School Students (master’s thesis). Changchun, China: Northeast Normal University.

[ref28] Liu C. (2015). A Study on the Relationship among academic Self-Efficacy, flow Experience in Examination and test Anxiety of middle school Students . [Master’s thesis]. Changsha, China: Hunan Normal University.

[ref29] Liu Y. (2021). The Study of the Relationship and Intervention Between Exam Anxiety, Academic Self-Efficacy and Academic Achievement of Students in Grade 3 of Junior Middle School. (master’s thesis). Kunming, China: Yunnan Normal University.

[ref30] Lohiya N. Kajale N. Lohiya N. Khadilkar A. Khadilkar V. Gondhalekar K. . (2021). Test anxiety among school-going children and adolescents, factors affecting and impact on quality of life: a multicenter study. Indian J. Pediatr. 88, 892–898. doi: 10.1007/s12098-021-03676-x, 33630222

[ref31] Ma X. Li Y. (2018). Survey on the anxiousness in examination and academic self-efficiency, and relationship between them among students in rural junior middle schools. Educ. Measur. Eval. 5, 51–56. doi: 10.16518/j.cnki.emae.2018.05.009

[ref32] Morony S. Kleitman S. Lee Y. P. Stankov L. (2013). Predicting achievement: confidence vs self-efficacy, anxiety, and self-concept in Confucian and European countries. Int. J. Educ. Res. 58, 79–96. doi: 10.1016/j.ijer.2012.11.002

[ref33] National Bureau of Statistics of China (2011) Division method for eastern, central, western, and northeastern China. Available online at: https://www.stats.gov.cn/zt_18555/zthd/sjtjr/dejtjkfr/tjkp/202302/t20230216_1909741.htm (Accessed August 21, 2026).

[ref34] Page M. J. McKenzie J. E. Bossuyt P. M. Boutron I. Hoffmann T. C. Mulrow C. D. . (2021). The PRISMA 2020 statement: an updated guideline for reporting systematic reviews. Syst. Rev. 10:1. doi: 10.1186/s13643-021-01626-4, 33781348 PMC8008539

[ref35] Pajares F. (1996). Self-efficacy beliefs in academic settings. Rev. Educ. Res. 66:543. doi: 10.2307/1170653

[ref36] Pekrun R. (2006). The control-value theory of achievement emotions: assumptions, corollaries, and implications for educational research and practice. Educ. Psychol. Rev. 18, 315–341. doi: 10.1007/s10648-006-9029-9

[ref37] Putwain D. Daly A. L. (2014). Test anxiety prevalence and gender differences in a sample of English secondary school students. Educ. Stud. 40, 554–570. doi: 10.1080/03055698.2014.953914

[ref38] Putwain D. W. Daly A. L. Chamberlain S. Sadreddini S. (2015). ‘Sink or swim’: buoyancy and coping in the cognitive test anxiety-academic performance relationship. Educ. Psychol. 36, 1807–1825. doi: 10.1080/01443410.2015.1066493, 37339054

[ref39] Putwain D. W. Gallard D. Beaumont J. Loderer K. von der Embse N. P. (2021). Does test anxiety predispose poor school-related wellbeing and enhanced risk of emotional disorders? Cogn. Ther. Res. 45, 1150–1162. doi: 10.1007/s10608-021-10211-x

[ref40] Qin T. Zhang J. Zhang Y. (2017). A correlational study on test anxiety and its relationship with academic self-efficacy among senior high school students. J. Lyuliang Teach. College 34, 15–16.

[ref41] Qiu T. (2023). The Effect of Mindfulness on Test Anxiety in Junior High School Students: The Mediating Role of Academic Self-Efficacy. (master’s thesis). Nanning, China: Nanning Normal University.

[ref42] Ren W. Liang R. (2024). Pressure of entrance examination, peer effects and family extracurricular education investment. J. Financ. Econ. 50, 154–168. doi: 10.16538/j.cnki.jfe.20230816.302

[ref43] Ren J. Shen D. (2025). The development, challenges, and reform orientation of the high school entrance examination system. China Exam. 12, 22–29. doi: 10.19360/j.cnki.11-3303/g4.2025.12.003

[ref44] Robson D. A. Johnstone S. J. Putwain D. W. Howard S. (2023). Test anxiety in primary school children: a 20-year systematic review and meta-analysis. J. Sch. Psychol. 98, 39–60. doi: 10.1016/j.jsp.2023.02.003, 37253582

[ref45] Rosenthal R. (1979). The file drawer problem and tolerance for null results. Psychol. Bull. 86, 638–641. doi: 10.1037/0033-2909.86.3.638

[ref46] Shangguan J. Du Y. (2012). The mediating role of academic self-efficacy in the relationship between test anxiety and learning engagement among senior high school students. Mental Health Educ. Primary Sec. Sch. 23, 10–13.

[ref47] Tian X. Ming H. Wei L. (2023). Test anxiety and academic burnout of high school students: the chain mediating role of academic self-efficacy and self-control. Scient. Expl. Educ. 41, 67–73.

[ref48] Valls M. Sessa G. Tardif E. Mascret N. (2025). Clusters of test anxiety in lower secondary students: a cross-cultural comparison between France and Switzerland. Front. Educ. 10:1682152. doi: 10.3389/feduc.2025.1682152

[ref49] Von der Embse N. Jester D. Roy D. Post J. (2018). Test anxiety effects, predictors, and correlates: a 30-year meta-analytic review. J. Affect. Disord. 227, 483–493. doi: 10.1016/j.jad.2017.11.048, 29156362

[ref50] Wang H. Huang L. (2014). The relationship between meta-worry and test anxiety of junior high school students: the mediating effects of academic self-efficacy 22, 769–771. doi: 10.13342/j.cnki.cjhp.2014.05.051

[ref51] Wu Y. Wong J. L. N. Song H. Zhou S. (2025). ‘The art of being governed’: unravelling teachers’ agency in reconciling academic burden within China’s high-stakes exam system. Eur. J. Educ. 60:4. doi: 10.1111/ejed.70317, 40046247

[ref52] Xiang H. (2013). A Study on the Relation Among Academic Self-Efficacy Resilience and Test Anxiety in High School Students. (master’s thesis). Changsha, China: Hunan Normal University.

[ref53] Xie Y. (2013). A study on test anxiety and its relationships with social support and academic self-efficacy among junior high school students. Academy. 11, 199–201.

[ref54] Yang L. (2010). A Study on Relationship Among Senior Students’ Test Anxiety and Time Management Disposition and Self-Efficacy (master’s thesis). Changchun, China: Northeast Normal University.

[ref55] Yang D. (2017). The Relationship Among Resilience, Academic Self-Efficacy, Test Anxiety and Academic Achievement in senior Students (Master’s thesis). Guiyang, China: Guizhou Normal University.

[ref56] Yang Q. (2022). Effect of academic self-efficacy on test anxiety for middle-school students: the chain mediating effect and its gender difference. Chin. J. Clin. Psychol. 30, 414–420. doi: 10.16128/j.cnki.1005-3611.2022.02.033

[ref57] Zhang Y. Li S. Yu G. (2019). The relationship between self-esteem and social anxiety: a meta-analysis with Chinese students. Adv. Psychol. Sci. 27, 1005–1018. doi: 10.3724/SP.J.1042.2019.01005

[ref58] Zhao J. (2006). Correlating Research on Self Efficacy, test Anxiety and Intellectual Achievement Responsibility of senior high school Students. (master’s thesis). Shijiazhuang, China: Hebei Normal University.

[ref59] Zhao N. (2014). A Study on the Relationship of parental Expectation, academic Self-Efficacy and academic Emotions among the Senior high school Students. (master’s thesis). Hohhot, China: Inner Mongolia Normal University.

[ref60] Zhong Y. (2021). The Influence of Parenting Style on Test Anxiety of Junior School Students: The Mediating Role of Academic Self Efficacy and The Moderating Effects of Gender. (master’s thesis). Jingzhou, China: Yangtze University.

[ref61] Zhou F. (2019). The Influence of Parental Rearing Patterns on Test Anxiety of Junior Middle School Students: The Mediating role of Self-Esteem and academic Self-Efficacy (master’s thesis). Dalian, China: Liaoning Normal University.

[ref62] Zhou C. (2024). Relationship Between Academic Self-Efficacy and Learning Burnout in Junior High School Students: The Mediating Effect of Test Anxiety and an Intervention Study. (master’s thesis). Beijing, China: Minzu University of China.

[ref63] Zhu Y. (2015). A meta-analysis of the relationship between self-efficacy and test anxiety among Chinese students. Chin. J. Sch. Health. 36, 527–531. doi: 10.16835/j.cnki.1000-9817.2015.04.018

