# Supplementary material for: The relationship between academic self-efficacy and test anxiety: a meta-analysis of Chinese secondary school students
Source: Front Psychol. 2026 Sep 23;17:1891963. doi: 10.3389/fpsyg.2026.1891963 (PMC13642300; doi:10.3389/fpsyg.2026.1891963)
Supplement: Supplementary file 1 [file Data_Sheet_1.PDF]

Table 1 Characteristics of the 30 included primary studies

Author, Publication year	Publication type	*r*	n	Quality of literature	Academic self-efficacy measurement instrument	Test anxiety measurement instrument	Age range	Percentage of female (%)	Grade level	Cronbach's α	Geographic distribution
Chen S et al, 2007	General journal	-0.400	3511	4	Liang Yushong version scale	Tian Bao version scale	16-18 years old	47.3%	10-12 grades	0.82(ASE) 0.914(TA)	Zhangzhou, Fujian province, China
Chen S et al, 2009	Core journals	-0.470	799	5	Liang Yushong version scale	Tian Bao version scale	Not reported	50.1%	10 grade	0.82(ASE) 0.914(TA)	Zhangzhou, Fujian province, China
Chen S, 2010	General journal	-0.488	544	4	Liang Yushong version scale	Tian Bao version scale	16-18 years old	42.1%	10-12 grades	0.817(ASE) 0.914(TA)	Zhangzhou, Fujian province, China
Cui K, 2022	Master's and doctoral Thesis	-0.329	952	6	Liang Yushong version scale	TAS	12-15 years old	Not reported	7-9 grades	0.881(ASE) 0.861(TA)	Guilin City, Guangxi province, China
Feng G, 2021	Master's and doctoral Thesis	-0.465	870	4	Liang Yushong version scale	TAI	16-18 years old	44%	10-12 grades	0.817(ASE) 0.81(TA)	Nantong, Jiangsu province, China
Gu M, 2020	Master's and doctoral Thesis	-0.245	1556	6	Liang Yushong version scale	TAS	14-18 years old	49.1%	10-12 grades	0.89(ASE) 0.86(TA)	Changsha, Leiyang, Hunan province, China
Guo D, 2016	Master's and doctoral Thesis	-0.301	548	5	Liang Yushong version scale	TAS	16-18 years old	69.89%	10-12 grades	0.855(ASE) 0.64(TA)	Baoding, Hebei province, China
Lei J,2019	General journal	-0.230	353	5	Liang Yushong version scale	TAS	16-18 years old	40.2%	10-12 grades	0.82(ASE) 0.64(TA)	Chuzhou, Anhui province, China
Lei W et al, 2021	SSCI	-0.110	552	7	Liang Yushong version scale	TAS	15-18 years old	49.6%	10-12 grades	0.88(ASE) 0.85(TA)	Shanxi province, China
Li J, 2020	Master's and doctoral Thesis	-0.244	6203	6	Liang Yushong version scale	Li Jiahao version scale	12-14 years old	47%	7-8 grades	0.868(ASE) 0.805(TA)	Henan province, China
Li X, 2022	Master's and doctoral Thesis	-0.450	655	5	Liang Yushong version scale	TAS	12-15 years old	49.5%	7-9 grades	0.87(ASE) 0.86（TA）	Taiyuan, Shanxi province, China
Liu C, 2015	Master's and doctoral Thesis	-0.310	406	4	Liang Yushong version scale	TAI	12-15 years old	57.4%	7-9 grades	0.827(ASE) 0.86(TS)	Hengyang, Hunan province, China
Liu T, 2012	Master's and doctoral Thesis	-0.429	302	5	Wang Zhenhong version scale	TAS	12-14 years old	51%	7-8 grades	0.910(ASE) 0.719(TS)	Baicheng, Jilin province, China

Table 1 (continued)

Author, Publication year	Publication type	*r*	n	Quality of literature	Academic self-efficacy measurement instrument	Test anxiety measurement instrument	Age range	Percentage of female (%)	Grade level	Cronbach's α	Geographic distribution
Liu Y, 2021	Master's and doctoral Thesis	-0.368	317	4	Liang Yushong version scale	TAS	Not reported	42.6%	9 grade	0.817(ASE) 0.62(TA)	Kunming, Yunnan province, China
Ma X et al, 2018	General journal	-0.221	533	6	Liang Yushong version scale	Zheng Richang version scale	12-15 years old	48.8%	7-9 grades	0.889(ASE) 0.910(TA)	Xingtai, Hebei province, China
Qin T et al, 2017	General journal	-0.364	214	4	Liang Yushong version scale	TAS	16-18 years old	55%	10-12 grades	Not reported	Changzhi, Shanxi province, China
Qiu T, 2023	Master's and doctoral Thesis	-0.359	713	5	Liang Yushong version scale	TAS	12-15 years old	46.8%	7-9 grades	0.88(ASE) 0.85(TA)	Nanning, Guangxi region, China
Shangguan J et al, 2012	General journal	-0.320	471	4	Hu Guiying version scale	TAS	16-17 years old	49.5%	10-11 grades	0.660(ASE) 0.884(TA)	Jiangsu province, China
Tian X et al, 2023	General journal	-0.454	337	4	Liang Yushong version scale	TAS	16-18 years old	45.7%	10-12 grades	0.891(ASE) 0.872(TA)	Wuhan, Hubei province, China
Wang H et al, 2014	General journal	-0.340	297	6	Wang Zhenhong version scale	TAS	12-15 years old	50.5%	7-9 grades	0.865(ASE) 0.899(TA)	Ningxia region, China
Xiang H, 2013	Master's and doctoral Thesis	-0.394	507	5	Liang Yushong version scale	TAS	16-18 years old	54.6%	10-12 grades	0.889(ASE) 0.64(TA)	Changde, Hunan province, China
Xie Y, 2013	General journal	-0.792	442	4	Ge Yan version scale	Zheng Richang version scale	12-15 years old	43.9%	7-9 grades	Not reported	Guangzhou, Guangdong province, China
Yang D, 2017	Master's and doctoral Thesis	-0.310	561	5	Liang Yushong version scale	TAS	Not reported	56.5%	12 grade	0.889(ASE) 0.817(TA)	Luoping, Guizhou province, China
Yang L, 2010	Master's and doctoral Thesis	-0.636	297	4	Liang Yushong version scale,	TAS	16-18 years old	79.7%	10-12 grades	0.891(ASE) 0.834(TA)	Hebei province, China
Yang Q, 2022	Core journals	-0.270	1013	7	Liang Yushong version scale	TAS	12-16 years old	56.6%	7-10 grades	0.85(ASE) 0.83(TA)	Guizhou province and Chongqing municipality, China
Zhao J, 2006	Master's and doctoral Thesis	-0.212	501	4	Zhao Jie version scale,	TAS	16-18 years old	53.3%	10-12 grades	Not reported	Dingzhou, Hebei province, China
Zhao N, 2014	Master's and doctoral Thesis	-0.105	264	6	Liang Yushong version scale	Zheng Richang version scale	14-17 years old	43.9%	7-9 grades	0.87(ASE) 0.89(TA)	Hohhot, Inner Mongolia region, China

Table 1 (continued)

Author, Publication year	Publication type	*r*	n	Quality of literature	Academic self-efficacy measurement instrument	Test anxiety measurement instrument	Age range	Percentage of female (%)	Grade leve	Cronbach's α	Geographic distribution
Zhong Y, 2021	Master's and doctoral Thesis	-0.340	3263	4	Liang Yushong version scale	TAS	12-15 years old	45.2%	7-9 grades	0.82(ASE) Not reported(TA)	Sichuan province, China
Zhou C, 2024	Master's and doctoral Thesis	-0.276	371	4	Liang Yushong version scale	TAS	12-14 years old	53.1%	7-8 grades	0.870(ASE) 0.64(TA)	Beijing, China
Zhou F, 2019	Master's and doctoral Thesis	-0.227	513	6	Liang Yushong version scale	TAS	12-15 years old	49.3%	7-9 grades	0.897(ASE) 0.856(TA)	Liaoning province, China

Note. *r*=effect size. n=sample size

Table 2 Full search strategies for each database

<table>
<tr><th>Database</th><th>User-entered search query</th><th>Search field & filters</th></tr>
<tr><td>Web of Science</td><td>Academic self-efficacy (Topic) and Test anxiety (Topic)</td><td>Smart Search (Topic field: title, abstract, keywords). Publication date: 2000-01-01 TO 2026-04-30. Note: Smart Search automatically performs semantic expansion. Full underlying Boolean syntax is not available for export.
The specific search records are as follows:
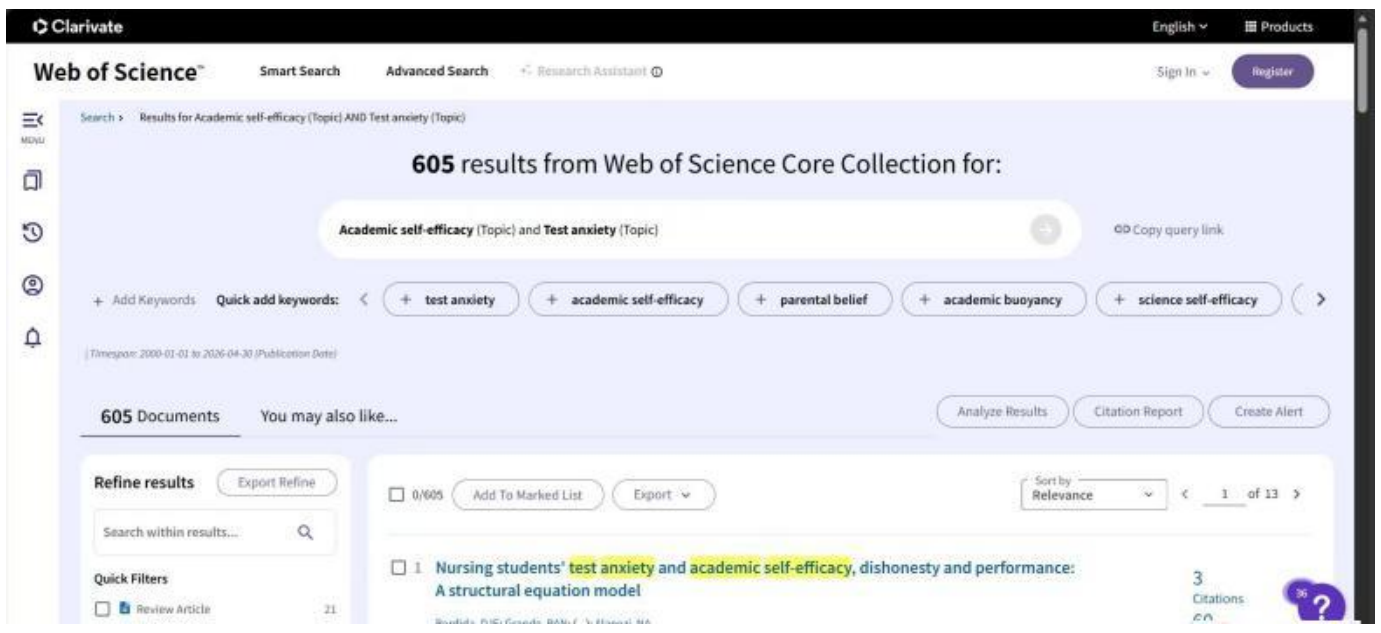
</td></tr>
<tr><td>CNKI</td><td>1.(学业自我效能感 + 学习自我效能感) * (考试焦虑 + 测试焦虑)
2.自我效能感 * (考试焦虑 + 测试焦虑)</td><td>Advanced Search (According to the key words) . Publication date: 2000-01-01 TO 2026-04-30.
The specific search records are as follows:
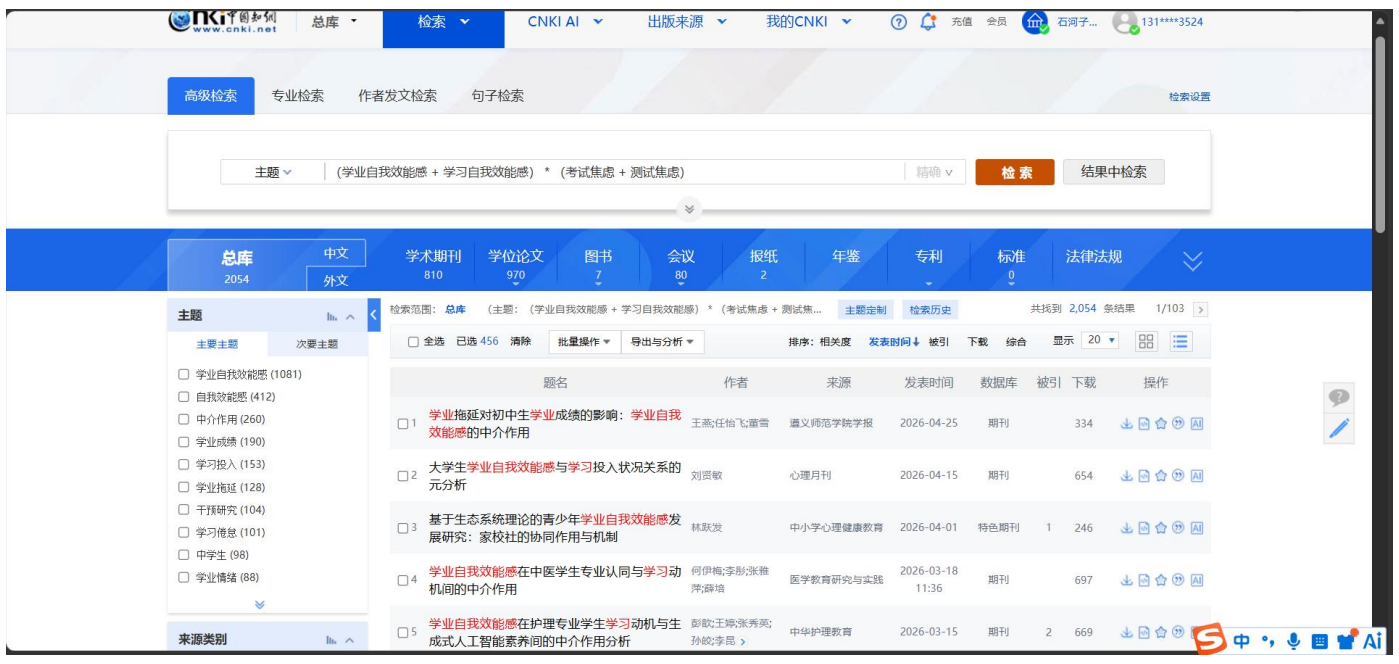
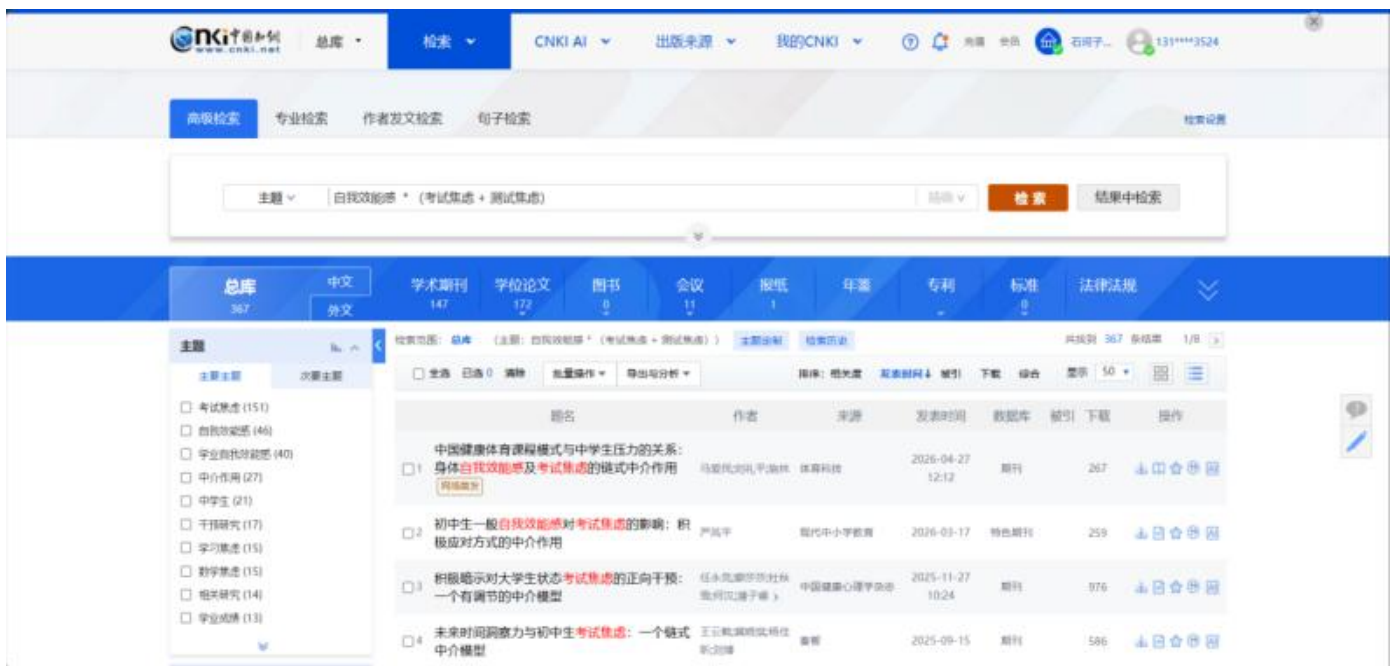</td></tr>
</table>

Table 3 Quality assessment scores for each included study

Author, Publication year	Item 1 score	Item 2 score	Item 3 score	Item 4 score	Total score
Zhou F, 2019	2	2	2	0	6
Zhou C, 2024	2	1	1	0	4
Zhong Y, 2021	2	2	0	0	4
Zhao N, 2014	2	2	2	0	6
Zhao J, 2006	2	2	0	0	4
Yang Q, 2022	1	2	2	2	7
Yang L, 2010	0	2	2	0	4
Yang D, 2017	1	2	2	0	5
Xie Y, 2013	2	2	0	0	4
Xiang H, 2013	2	2	1	0	5
Wang H et al, 2014	2	2	2	0	6
Tian X et al, 2023	1	1	2	0	4
Shangguan J et al, 2012	1	2	1	0	4
Qiu T, 2023	1	2	2	0	5
Qin T et al, 2017	2	2	0	0	4
Liu Y, 2021	1	2	1	0	4
Liu T, 2012	1	2	2	0	5
Liu C, 2015	1	1	2	0	4
Li X, 2022	1	2	2	0	5
Li J, 2020	2	2	2	0	6
Lei J,2019	2	2	1	0	5
Guo D, 2016	2	2	1	0	5
Gu M, 2020	2	2	2	0	6
Feng G, 2021	1	1	2	0	4
Cui K, 2022	2	2	2	0	6
Chen S et al, 2009	1	0	2	2	5
Chen S et al, 2007	2	0	2	0	4
Chen S, 2010	2	0	2	0	4
Ma X et al, 2018	2	2	2	0	6
Lei W et al, 2021	1	2	2	2	7

Note. For items assessing internal-consistency reliability, the arithmetic mean of reliability coefficients from the two core measurement tools (academic self-efficacy and test anxiety scales) was used for scoring. For studies whose journals are indexed in multiple databases, scores for journal tier (item 4) were assigned based on the highest journal rank.

Table 4 Complete results of leave-one-out sensitivity analysis

Excluded Study（Author, Publication year）	Pooled *r*	Upper and lower limits of the95% confidence interval	*Z*-Value	*p*-Value
Zhou F, 2019	-0.364	[-0.410, -0.315]	-13.607	0.000
Zhou C, 2024	-0.362	[-0.409, -0.313]	-13.518	0.000
Zhong Y, 2021	-0.360	[-0.410, -0.308]	-12.689	0.000
Zhao N, 2014	-0.367	[-0.413, -0.319]	-13.925	0.000
Zhao J, 2006	-0.364	[-0.411, -0.316]	-13.652	0.000
Yang Q, 2022	-0.362	[-0.410, -0.313]	-13.393	0.000
Yang L, 2010	-0.349	[-0.394, -0.302]	-13.575	0.000
Yang D, 2017	-0.361	[-0.408, -0.312]	-13.404	0.000
Xie Y, 2013	-0.338	[-0.375, -0.300]	-16.413	0.000
Xiang H, 2013	-0.358	[-0.405, -0.309]	-13.326	0.000
Wang H et al.	-0.360	[-0.407, -0.311]	-13.441	0.000
Tian X et al.	-0.356	[-0.403, -0.307]	-13.348	0.000
Shangguan J et al.	-0.361	[-0.408, -0.312]	-13.413	0.000
Qiu T, 2023	-0.359	[-0.407, -0.310]	-13.294	0.000
Qin T et al., 2017	-0.359	[-0.406, -0.310]	-13.445	0.000
Ma X et al.	-0.364	[-0.410, -0.315]	-13.623	0.000
Liu Y, 2021	-0.359	[-0.406, -0.308]	-13.402	0.000
Liu T, 2012	-0.357	[-0.404, -0.308]	-13.361	0.000
Liu Z, 2015	-0.361	[-0.408, -0.312]	-13.448	0.000
Li X, 2022	-0.356	[-0.403, -0.307]	-13.315	0.000
Li J, 2020	-0.363	[-0.411, -0.313]	-13.218	0.000
Lei J, 2019	-0.363	[-0.410, -0.315]	-13.618	0.000
Guo D, 2016	-0.361	[-0.408, -0.312]	-13.424	0.000
Gu M, 2020	-0.363	[-0.411, -0.314]	-13.411	0.000
Feng G, 2021	-0.355	[-0.402, -0.307]	-13.341	0.000
Cui K, 2022	-0.360	[-0.408, -0.311]	-13.266	0.000
Chen S et al.	-0.355	[-0.402, -0.307]	-13.351	0.000
Chen S et al.	-0.358	[-0.407, -0.307]	-12.813	0.000
Chen S, 2010	-0.354	[-0.401, -0.306]	-13.368	0.000
Lei W et al.	-0.367	[-0.413, -0.320]	-14.055	0.000
All Studies	-0.359	[-0.405,-0.312]	-13.722	0.000

Note. Pooled *r*=pooled correlation coefficient. k=number of included studies. *Z*=test statistic for evaluating whether pooled *r* differs significantly from zero. *p*=corresponding *p*-value for the significance test of pooled *r*.

Table 5 Database links and accession numbers for included studies

Author	Title	Database links or accession numbers
LI X, 2022	The influence of parental psychological control on testanxiety of junior high school students: the chain mediating effect of self-esteem and academic self-efficacy	https://kns.cnki.net/kcms2/article/abstract?v=LT90ZeEa8Uy7l1UbB68zDfXhfHnq4DoD7eRkrZvwxm4epsXGP3IoBpmBnJWZ9DvkndBgEbj5wxq_VkttTD0Kv0E4CSSYBjpwCi7GDNnOOcCF1cvaRwaw3TCuQnV27GbEGCMzOcIZDbq1_Zu9N0XqEblL3PV8rbodeAjdCpBJuk_saMz6KXF5sNl51dQS5yEy&uniplatform=NZKPT&language=CHS
Yang Q, 2022	Effect of Academic Self-efficacy on Test Anxiety for Middle-school Students:The Chain Mediating Effect and Its Gender Difference	https://kns.cnki.net/kcms2/article/abstract?v=LT90ZeEa8Uy31DqrZ0U3vg5jkeUsIRIO5RmMxhjDFmGQW4uFlCAzil8pNaQwVRc3Wl-rVuD2HuYqY2ZlyxvuSLeaRD-zhn-LvR_ixaqktEb8ro1GRbsBIClkYSZh2doZOumsEqIrVIj1F-wryvUglQWvLmEFXxHoHLO6LN6ZM8XdQ0CW9_A0jQ==&uniplatform=NZKPT&language=CHS
Liu Y, 2021	The study of the Relationship and Intervention between Exam Anxiety,Academic Self-efficacy and Academic Achievement of students in grade 3 of Junior middle school	https://kns.cnki.net/reader/flowpdf?invoice=KmIlaFJ%2BI7gkh9hlDG%2Bxt0iLXEYIaY7zHwDx9R5jr8HMZ%2FRb7RR%2ForECltxDnlJtH8jh4kEcdzIZ2elcR6zOw7lMLvGClROFNZA5IUitQTPW8dq9Ft4fYIxgoBztkSCmodfeFXlAEMxT6a%2F0B%2BQcjI4Tk1C3AwxOj8RYMlnFvIs%3D&platform=NZKPT&sourcetype=nxgp&product=CMFD&filename=1021620995.nh&tablename=cmfd202102&type=DISSERTATION&scope=trial&cflag=overlay&dflag=pdf&pages=&language=CHS&trial=&nonce=2F0C13FEB88F417B8D4E72D61F5E9ED2
Zhong Y, 2021	The influence of parenting style on test anxiety of juniorschool students: the mediating role of academic selfefficacy and the moderating effects of gender	https://kns.cnki.net/kcms2/article/abstract?v=LT90ZeEa8UwSxKcyZSK-unwxv9EGsWN8W70LWzCaIL4QIGWxzz-7-rYHSHLyaFt5MasVuAD5awuw_rkloeyQDHH7F4FvSyGkF1aJrYfnKgoZ5_KXH-kNcoP2K2m7l-Bnhc0jlvgTbSAjidzCia84MvKzVnUH8bSseV68hASaTYxQIyy30htJ206Xc9wLQPjF&uniplatform=NZKPT&language=CHS
Li J, 2020	The Relationship Between Junior Middle School Student's Test Anxiety and Academic Achievement: The Mediating Effect of Academic Self-efficacy	https://kns.cnki.net/kcms2/article/abstract?v=LT90ZeEa8UxE8SsTvcEHLglvGhQo31Q0TZhcjpgtmEGtlTSWb94JhsTgudCiv9hyaBiXYv5JXMiKtZwmheyO0duGehbseCtLZ97DoGMYipR2Oh7r1rlMe3SBrwqm2zxUYxpN-ShiI40H4GYH_frCcuVQXe5rU03eoVd-02qKLmwpx-5DfMpLSNCRaibIgp_V&uniplatform=NZKPT&language=CHS
Lei J, 2019	A Study on the Correlation between Academic Self-Efficacy and Test Anxiety among High School Students	https://kns.cnki.net/kcms2/article/abstract?v=LT90ZeEa8UzKKIbeLhjh7RPv3GO2ShntYVwtX3kGiMxHBH3XODMlzU0J_jPIS0aWzr19t74hHxwbDhoMP0pQkfsrKWW7E4RKBf8SYK8EgJammdkeVym2kqPvZPmkMwWkMHXvNqRK57t-SXLVs4vBjH0GjwtH2JTqfRUAzVSeBpsCWWOJ5Ukasw==&uniplatform=NZKPT&language=CHS
Zhou F, 2019	The Influence of Parental Rearing Patterns on Test Anxiety of Junior Middle School Students: The Mediating Role of Self-esteem and Academic Self-efficacy	https://kns.cnki.net/kcms2/article/abstract?v=LT90ZeEa8Uxpk4CYqjCPsySwHneTmVd5ds5T9oDCdTNuMDp8sxfWJ-uff3i1tNfgenvMbxf4vJ3hBTYYuf5zSrxZmYw3raE8diFA-J3Dt-4pz3rSYxWHVvWt4uWWFRE56QnHHQJ2oXZ1lTcL1mHAfukvHTdmZzVS51rscesW4fBh4Mdt2g9_nenDYnixkLfd&uniplatform=NZKPT&language=CHS
Ma X et al, 2018	Exam Anxiety, Academic Self-Efficacy and Their Relationship among Rural Junior High School Students	https://kns.cnki.net/kcms2/article/abstract?v=LT90ZeEa8Uxj-4X6gym5in66C8LQ2UgeFI4hWpu8kV4ZVSBJm8hSSSb896jPbFU-JeFaUzBUFFH2tFqxr_poSeMPfqq3RfzmhRUNce8_ssENO-wEfliGeTDNZho1rQDpwkan_gZcRM_RIO1ahwedjYb1xC3Nh-pN4YA1sKiu6X8-XoCcrjkc4A==&uniplatform=NZKPT&language=CHS
Guo D, 2016	A Research on the Relationship among Academic Self-efficacy, Text Anxiety and Learning Burnout of the High School Students	https://kns.cnki.net/kcms2/article/abstract?v=LT90ZeEa8UxEMeebTN1MknrNak3_u6RSBtsp46jhBuGP9XqmMszBwlyGXS6mlvxHYhtCVk8mFNUOgtmxqmrqbnJAb75NRI5biACx-AfO4GC-3VShl0dJILx6tPFJGPMexc7B4klUmyjBRgF7Xa5xeRcJe5Llmlt05yHIf3fMUFxGf57LYzKN8w9eakg2uzAb&uniplatform=NZKPT&language=CHS
Liu C, 2015	A Study on the Relationship among AcademicSelf-efficacy, Flow Experience in Examinationand Test Anxiety of Middle School Students	https://kns.cnki.net/kcms2/article/abstract?v=LT90ZeEa8UwRMzTzQ5eFr6Ztw2_-gJgWfkRqgqchmNRoRNYVs9xbJCkll4Ac24Z7yZmF57YwLe90-edh6J0eytFQlvI7N6mS4xViKuoyJ3ftOCx5VPp_CA3_djh6qayv2mvg_eTh5t3ACkfG76G1Vp5R-wQCrJ0a4Gds99Dovs73jygtTuvo2S7-WgNRL1ws&uniplatform=NZKPT&language=CHS
Zhao N, 2014	A study on the relationship of parental expectation,academic self-efficacy and academic emotionsamong the Senior high school students	https://kns.cnki.net/kcms2/article/abstract?v=LT90ZeEa8UwA6jjWq8VZ5b5N-HDlLfIzRqJLQpiF5aoBjAtuzzfAPM_Sa8bSzxgZTR949-pR0FqIhlqUQpUcOo_0kgVCYPpGkVvXCR5yM2efY7N1SBNLdDvfINTbwdYjuVK263T9jiojBFike6pnno-KApF7RCWHvO5dUoDmaytlqe1eH0Yn02SFsW8uIC_R&uniplatform=NZKPT&language=CHS

Table 5 (continued)

Author	Title	Database links or accession numbers
Wang H et al, 2014	The Relationship between Meta-worry and Test Anxiety of Junior High School Students: The Mediating Effects of Academic Self-efficacy	https://kns.cnki.net/kcms2/article/abstract?v=LT90ZeEa8UzDw27M5lNJAeX2DLBUFtxUyKQU3UmPewJkyyqnHI4Dz70Vw3DIIfFPILj2XwsUgAry2S9DxyGkw7IsCPHGqHLybOwauJI_kWu2fP82ftSqRk4LMRS7OgAkKuAfUtM13plz_Sh6PL2NJv3wE_QxFJJcNPRbS0d6BHkFC4-je2bOuA==&uniplatform=NZKPT&language=CHS
Xiang H, 2013	A study on the Relation among AcademicSelf-efficacy Resilience and TestAnxiety in High School Students	https://kns.cnki.net/kcms2/article/abstract?v=LT90ZeEa8Uz0P-R8TAZO8wmUTx5Zk21k9nm6sARhjebF0clf_BpxZONLFkVLN0Bo30rpEt5bLlz3wb-UIl8DNozAaLp7Mp1MXqnwAQIXhz04PkVFv9RjaaaeWApCiX1JH_6KYk_KZ01yEj5OXo4Ro-Q_7chrNxWCUrUl-ZBZv8qptFf7cPAvMBjNiDiGrvnU&uniplatform=NZKPT&language=CHS
Xie Y, 2013	Research on the Examination Anxiety of Middle School Students and Its Relationship with Social Support and Academic Self-efficacy	https://kns.cnki.net/kcms2/article/abstract?v=LT90ZeEa8UwtsJ1yMGigMxcpvYAvDfx8KTmpnF2BlxcTbX1NAElUIvhBhSLSuixQM_aoGW0CckVBNTCAGekIWmf4V4S2rCvL6H4o5PqEKPb8JsxN8HRP-oeOOMs3bzfvY3JXZd3nuu8yRcNnxsSPvOTJNyUURCiQk3NhPpqY8BbIMHZjVrlIdQ==&uniplatform=NZKPT&language=CHS
Shangguan J et al, 2012	The mediating role of high school students' self-efficacy in the relationship between test anxiety and learning engagement	https://kns.cnki.net/kcms2/article/abstract?v=LT90ZeEa8Uw7Cuci5OBNV3oQ_lCFA9ni7bqaceovArFsasuZXbnx9RU77H739iHZv1cS_5e7p3SlKPB2IFsoTMmhA1ycpvFj95ro8r4y8rfULlfrdZYhdBp8sI9bZajr_buMfTELIoreOLHLS98YlkIivIy5gBveR5Nh9kPjX7679fGiu4usFg==&uniplatform=NZKPT&language=CHS
Liu T, 2012	A Study on the Relationship of Academic Adaptability,TestAnxiety, Academic Self-Efficacy and Academic Achievementamong Junior Middle School Students	https://kns.cnki.net/kcms2/article/abstract?v=LT90ZeEa8Uzx4PPDzixjA6sKbzFETxbLhgNym_BE17cJ-7zsaFbpVxaTdvJKmzkd3CsD6JdBXTKtOKliQkIYV37sM8_slJLwQ-900FTqz8lcu3SDUgUws20Di5UeZ0AvTx7m1Gja8BxNFL-C2-AnD5ew6QOs2Wd70hzReF7rAkNjVDPC8cjs-nnk4Msv9By7&uniplatform=NZKPT&language=CHS
Chen S, 2010	The relationship between the behavioral types of high school students, academic self-efficacy, and test anxiety	https://kns.cnki.net/kcms2/article/abstract?v=LT90ZeEa8Uwtw7XI37EDVyEqDluUZ59Ca0S2C2Z4OH0hmiYBu0izD6C-cHLpOhl8DIIn-Pdu4P0pafjWTQof2AGtsvUwvp-ULihVfF-EASKULee6k-C3zeBq3J28ZXCHUs0UfPfGRP6bFjboYMCTd4ZOwjxGuKsIETMXyzEb8hUg6xRkEUEt-g==&uniplatform=NZKPT&language=CHS
Chen S et al,2007	High School Students' Test Anxiety and Its Relationship with Academic Self-Efficacy	https://kns.cnki.net/kcms2/article/abstract?v=LT90ZeEa8UwJ8h9FKRnhRuk_x83HbqH0hbiy5cYsgvxwqDEPkEy4hXtWUhBO4QvcnI4JKdEqfoLQ3PGYurdCRKBWlLALUTE7r1syHc62MDu_IuiDS6eCgDrGcPMiE6uhCHXKDlEV5VKeoSye0qvMVf-SGC5tWAo3eMOSYpe6OC5rEzJ5u5qKKA==&uniplatform=NZKPT&language=CHS
Zhao J, 2006	Correlating Research on Self Efficacy, Test Anxiety and Intellectual Achievement Responsibility of Senior High School Students	https://kns.cnki.net/kcms2/article/abstract?v=LT90ZeEa8UwtPOkAInZ7OmFDL1esfabgD9CxxfPuie42xXTBfvoNtEr4UW5Cd0wJql6spu7KUi4ZBlChG8cJGiGOCDZQR0zlOYEv-dg44qeXY6axND6-kiRRuAaypcN2GMqC6IDDXDTMHaLQDsdDeKMw09e3QnQ_4OR0PKm8m-VHszKFcjuCPg==&uniplatform=NZKPT&language=CHS
Zhou C, 2024	The Relationship between Junior High School Students' Academic Self-Efficacy and Learning Burnout: The Mediating Effect of Test Anxiety and the Intervention Study	https://kns.cnki.net/kcms2/article/abstract?v=LT90ZeEa8UxQ7O6AamZZ04Hx68zjuij9g5CpPzSHxzjdxmeoppynCukq5GZ7vS7UoStniVlozDr9NikJVY2uRjtQJodWpr6DldvldEgr6I3wGMvMlyewtl42ifwArlcMFsiRKdg4HPmHmil96Jh20QrsobTuKNQG9lets05eU2ZZft6f2Rp3eUM7Xz2cr4GW&uniplatform=NZKPT&language=CHS
Tian X et al, 2023	High School Students' Test Anxiety and Academic Burnout: The Chain Mediating Role of Academic Self-Efficacy and Self-Control	https://kns.cnki.net/kcms2/article/abstract?v=LT90ZeEa8UznbHKryTRdofPdhVbZulLSOMcO7UDpOfuA1l_tQ0X1CDeUXv7rSXqMl_DzNU_dsudebveSUzajCHCYc__qRp8_EXZbNvnsL0nPzD6HG1CEBbvayvTva4qO2l_QDgfWm0428NQrS3hXJVo1RKUsGNywF_XEFaLFG0q8pbMY1WsBOQ==&uniplatform=NZKPT&language=CHS
Qiu T, 2023	The Effect of Mindfulness on Test Anxiety in Junior HighSchool Students: The Mediating Role of AcademicSelf-Efficacy	https://kns.cnki.net/kcms2/article/abstract?v=LT90ZeEa8UyFn3lScZon6Hwc_KJE80Uws9ED5TFJwhQvhySF0l-qr6EJm-fKMduiyQ84r4MY46Cbdq_ws3pOXnRYJFk5SRQ7ERFeXAgSRaE2dXMdTPNmSkgQkEv61kNCRQsm4AAmweYd93Nls4SXnD0Z-rY6QY0qRBYu5lMH4SCwemg-GhbNLBXAJccCkiU0&uniplatform=NZKPT&language=CHS
Feng G, 2021	The Relationship between Psychological Flexibility andTest Anxiety of Senior High School Students<br>----Mediating Role of Academic Self-efficacy	https://kns.cnki.net/kcms2/article/abstract?v=LT90ZeEa8UwpNwBTOHncXrgSXsR4MWT2qnOQuhTyFf7Yk6FA-zGi0VBVeeZM5IHg5oTMWe98W41tV0aBMgbKs3pv0SiVwEVfhSXTo3e_g8vaKcwKYEJ43n0N1zFzupd-p5_ZU7KCBftk23HXTvcfLLD2crsc8EWClRpOfm0mzFPdC3twJ9wU2JU5Tm1lgCLc&uniplatform=NZKPT&language=CHS

Table 5 (continued)

Author	Title	Database links or accession numbers
Gu M, 2020	The influence of parenting style of senior high school students on test anxiety: the mediating effect of learning self-efficacy	https://kns.cnki.net/kcms2/article/abstract?v=LT90ZeEa8UyjnzVZ6OiEA3IZI1P2UtuzSkYm4k1AtFTCZcBMs38Tp5P7OPSP3S8HSertgx0JDm6w9X7wq58CAq30_FIR4TyLe4GGCSAS3WMXPx6UnJWf_xUNWLU2s165o1E8NCKS0bc0bqM-TXI16hWPjFHHw41ergFLX5aPRXwhZvbRAcLWKTV-8nGgtafZ&uniplatform=NZKPT&language=CHS
Qin T et al, 2017	A Study on High School Students' Test Anxiety and Its Correlation with Academic Self-Efficacy	https://kns.cnki.net/kcms2/article/abstract?v=LT90ZeEa8Uz4zWA1CR59azoqgb8wJIPB0ARFRqBreQfx0KWJEbNZ4xO994h18vGCbBdfXsbrAOpalqy-Io8ePhiLTgWyp0c74hV4F03eqMDlKzhd-IuzQovz4c3ihB63ql_bM1_gTNQa4jXNFEHwD6Ra_wF_-g7J3x8zXFLzcBG2Fn33dwOB4Q==&uniplatform=NZKPT&language=CHS
Yang D, 2017	The Relationship among Resilience,Academic Self-efficacy, Test Anxiety and Academic Achievement in Senior Students	https://kns.cnki.net/kcms2/article/abstract?v=LT90ZeEa8UxCSzlOt4L3qq-CpqRjcYv20IH1kIZdzORQdT6b3sZlgBR_Ms5ssoPqdNFqnKYk4l9CFWOd34CMeSZLUcwfcVJ-wZxVgWJFyz2akfbUMigm-tXk932BTlMO4DJ2SwlPHPpttcLNIQsyEe06ed9dNSr9ndoa1Yf9y927pm5ILU1T6IK-C4peyst2&uniplatform=NZKPT&language=CHS
Yang L, 2010	A Study on Relationship among Senior Students'Test Anxiety and Time Management Disposition and Self-efficacy	https://kns.cnki.net/kcms2/article/abstract?v=LT90ZeEa8Uxihf2Kc1udsGykI_kohdquezdd2VRyPXU8hepom2u4tElXLvjPoIbJsojjPDwlk1Q_J68ybPpx4_ysiGB2NR1vbkDcWsVfV9oVwkvXGfnbUyIlMAGxqvxymoU37-uew_gDhgAAfoo7evjzWzkgmks780_IWeQ_xUStHszuD1YYvg==&uniplatform=NZKPT&language=CHS
Chen S et al, 2009	The relationship between exam anxiety and academic performance: A moderated mediating effect	https://kns.cnki.net/kcms2/article/abstract?v=LT90ZeEa8Uw092xy6VMTz2f1gucAeuRyS6LJfu730U0w83TwkgE78ZEU_2y6pGcNaa7K__PXtANDfY4QJih7wx-OL28SOB7vZEOssk7KkjgnXP1SJCApKhYL45A9hruCRlQj-HG9d9f39-0_4qzVPt8ZnDeKpfMCzIIFFZ7O1yBwX0kfQpVrvA==&uniplatform=NZKPT&language=CHS
Lei W et al	Academic self-efficacy and test anxiety in high school students: A conditional process model of academic buoyancy and peer support	https://sage.cnpereading.com/doi/10.1177/01430343211039265
Cui K, 2022	The Impact of Middle School Students' Academic Self-Efficacy on Test Anxiety: The Role of Negative Evaluation Anxiety and Core Self-Evaluation	https://kns.cnki.net/kcms2/article/abstract?v=LT90ZeEa8UwWijsjY_XfcGQwUtsNOpiH24WashekNbA9CMv-sk4DjABPFT-pDeHNc_mNpcgF6zpB0HtSzImqT2y6pFXmM7mN2jUi6AlbWb4PxGpcDk-oFi80S6v81HgDkwXosl16--NdyIzsHavwZVo_xzKtzJCaFn__5XnwGT_aARovT77sxIH-nR4egyRE&uniplatform=NZKPT&language=CHS

Table 6 Sources of measurement instruments

Instrument name	Author (Compiler)	Reference sources
Academic Self-Efficacy Questionnaire (ASEQ)	Wood	Wood, R.E., Locke, E.A. (1987). The relation of self-efficacy and grade goals to academic performance. Educational and Psychological Measurement. 47:4, 1013-1024. doi: 10.1177/0013164487474017
Academic Self-Efficacy Scale (ASE)	Chemers	Chemers, M.M., Hu, L., Garcia, B.F. (2001). Academic self-efficacy and first year college student performance and adjustment. Journal of Educational Psychology. 93:1, 55-64. doi: 10.1037/0022-0663.93.1.55
Bian Yufang version scale	Bian Yufang	Bian, Y.F. (2004). The development of a learning self-efficacy scale. Psychological Science. 27:5, 1218-1222. doi: 10.16719/j.cnki.1671-6981.2004.05.055
Ge Yan version scale	Ge Yan	Ge, Y. (2008). A correlating study on relations among learning stress, test anxiety, self-efficacy, and learning strategy of junior students. [Master's thesis]. Northeast Normal University.
Hu Guiying version scale	Hu Guiying	Hu, G.Y., Xu, B.H. (2002). A study on the relationship among learning attribution, self-efficacy, learning strategy and academic achievement of secondary school students. Psychological Science. 25:6, 757-758. doi: 10.16719/j.cnki.1671-6981.2002.06.041
Liang Yushong version scale	Liang Yushong	Liang, Y. S. (2000). A study on achievement goals, attribution styles, and academic self-efficacy of college students. [Master's thesis]. Central China Normal University.
Morgan-Jinks Student Efficacy Scale (MJSES)	Jinks	Jinks, J., Morgan, V. (1999). Children's perceived academic self-Efficacy: An inventory scale. The Clearing House: A Journal of Educational Strategies, Issues and Ideas. 72:4, 224-230. doi: 10.1080/00098659909599398
Motivated Strategies for Learning Questionnaire (MSLQ)	Pintrich	Pintrich, P.R., De Groot, E.V. (1990). Motivational and self-regulated learning components of classroom academic performance. Journal of Educational Psychology. 82:1, 33-40. doi: 10.1037/0022-0663.82.1.33
the College Academic Self-Efficacy Scale (CASES)	Owen	Owen, S. V., Froman, R. D. (1988). Development of a college academic self-efficacy scale. Paper presented at the 1988 annual meeting of the National Council on Measurement in Education, New Orleans, LA. https://files.eric.ed.gov/fulltext/ED298158.pdf.
Wang Zhenhong version scale	Wang Zhenhong	Wang, Z.H. (1999). Research on the relationship between academic self-efficacy and academic achievement of junior high school students. Psychological Development and Education. 15:1, 39–43.
Zeng Xinghua version scale	Zeng Xinghua	Zeng, X. H. (2008). Senior school students' academic self-efficacy and the relationship among academic self-efficacy, coping styles and mental health of senior school students. [Master's thesis]. Fujian Normal University.
Zhao Jie version scale	Zhao Jie	Zhao, J. (2006). Correlating research on self efficacy, test anxiety and intellectual achievement responsibility of senior high school students. [Master's thesis]. Hebei Normal University.

Table 6 (continued)

Instrument name	Author (Compiler)	Reference sources
Cassady version scale	Cassady	Cassady, J. C., Johnson, R. E. (2002). Cognitive test anxiety and academic performance. Contemporary Educational Psychology. 27:2, 270-295. doi:10.1006/ceps.2001.1094
Li Jiahao version scale	Li Jiahao	Li, J. H. (2020). The relationship between junior middle school student's test anxiety and academic achievement: The mediating effect of academic self-efficacy. [Master's thesis]. East China Normal University.
Test Anxiety Inventory (TAI)	Spielberger	Spielberger, C.D. (2010). Test anxiety inventory. The Corsini Encyclopedia of Psychology. 1-1. doi: 10.1002/9780470479216.corpsy0985
Test Anxiety Scale (TAS)	Sarason	Sarason, I.G., Sarason, B.R. (1990). Test anxiety. Handbook of Social and Evaluation Anxiety. 475-495. doi: 10.1007/978-1-4899-2504-6_16
Tian Bao version scale	Tian Bao	Tian, B., Guo, D.J. (2004). The basic model of test anxiety influencing test performance. Psychological Science. 27:6, 1360-1364. doi: 10.16719/j.cnki.1671-6981.2004.06.021
Wang Caikang version scale	Wang Caikang	Wang, C.K., Liu, Y. (2000). Correlation among general self-efficacy, trait anxiety, state anxiety and test anxiety. Chinese Journal of Clinical Psychology. 8:4, 229-230.
Zheng Richang version scale	Zheng Richang	Wu, J. J., Li, Y. W., Zheng, R. C. (2007). A study on the influential factors of test anxiety of senior students in high school. China Journal of Health Psychology. 15, 1019-1021. doi: 10.13342/j.cnki.cjhp.2007.11.029
